# Supplementary material for: KAT5 regulates neurodevelopmental states associated with G0-like populations in glioblastoma
Source: Nat Commun. 2025 May 9;16:4327. doi: 10.1038/s41467-025-59503-w (PMC12064679; doi:10.1038/s41467-025-59503-w)
Supplement: Supplementary file 1 — Supplementary Information [file 41467_2025_59503_MOESM1_ESM.pdf]

Supplementary Information

KAT5 regulates neurodevelopmental states associated with G0-like populations in glioblastoma

Anca B. Mihalas[1], Sonali Arora[1], Samantha A. O'Connor[2], Heather M. Feldman[1], Christine E. Cucinotta[3,4], Kelly Mitchell[1], John Bassett[5], Dayoung Kim[4], Kang Jin[6], Pia Hoellerbauer[1], Jennifer Delegard[7], Melissa Ling[8], Wesley Jenkins[8], Megan Kufeld[1], Philip Corrin[1], Lucas Carter[1], Toshio Tsukiyama[4], Bruce Aronow[6], Christopher L. Plaisier[2], Anoop P. Patel[9,10,11]*, and Patrick J. Paddison[1,8]*

[1]Human Biology Division, Fred Hutchinson Cancer Center, Seattle, WA 98109; [2]School of Biological and Health Systems Engineering, Arizona State University, Tempe, AZ 85281; [3]College of Arts and Sciences, Department of Molecular Genetics, Ohio State University, Columbus, OH 43210; [4]Basic Sciences Division, Fred Hutchinson Cancer Center, Seattle, WA 98109; [5]Department of Medicine, Karolinska Institute, Huddinge, Sweden; [6]Division of Biomedical Informatics, Cincinnati Children's Hospital Medical Center, Cincinnati, OH 45229; [7]Department of Neurosurgery, University of Washington, Seattle, WA 98195; [8]Molecular and Cellular Biology Program, University of Washington, Seattle, WA 98195; [9]Department of Neurosurgery, Duke University, Durham, NC 27710; [10]Preston Robert Tisch Brain Tumor Center, Duke University, Durham, NC 27710; [11]Center for Advanced Genomic Technologies, Duke University, Durham, NC 27710.
*To whom correspondence should be addressed: Anoop Patel (anoop.patel@duke.edu) or Patrick Paddison (paddison@fredhutch.org).

Supplementary Figures

Supplementary Figure 1: Data in support of **Figure 1**, including G0 reporter activity and supporting data for G0-trap screen.

Supplementary Figure 2: *KAT5* inhibition triggers G0-like state in GSCs in *vitro*, in support of **Figure 1**.

Supplementary Figure 3: *KAT5* inhibition triggers G0-like state in GSCs in *vitro*, in support of **Figure** .

Supplementary Figure 4: scRNA-seq filtering and data quality evaluation for **Figure 2**.

Supplementary Figure 5: Single cell gene expression analysis of KAT5 KO in GSC-0827 cells *in vitro*, in support of **Figure 2**.

Supplementary Figure 6: Single cell RNA-seq data analysis associated with **Figure 3**.

Supplementary Figure 7: scRNA-seq filtering and data quality evaluation for scRNA-seq data for GSC-0827 tumor reference, in support of **Figure 4**.

Supplementary Figure 8: Data associated with **Figure 4**, including scRNA-seq data for GSC-0827 tumor reference.

Supplementary Figure 9: Data associated with **Figure 4** and **Supplementary Figure 8**, including a dot plot top differentially expressed genes from scRNA-seq GSC-0827 tumor data.

Supplementary Figure 10: Data associated with **Figure 4** and **Supplementary Figure 8.** The full heatmap of neurodevelopmental gene expression profiles from **Supplementary Figure 8.**

Supplementary Figure 11: Data associated with **Figure 4**, including scRNA-seq data for GSC-464T tumor reference.

Supplementary Figure 12: Data in support of **Figure 5**, including scRNA-seq analysis and CUT&TAG analysis of GSC-0827 Clone 13 tumors.

Supplementary Figure 13: Data in support of **Figure 6**, including additional data for in vivo KAT5 KO survival gain and GSC invasion assays.

Supplementary Figure 14: Data in support of **Figures 6** and **7**, including association of Neural G0 gene expression with glioma grade, IDH mutation status, and survival.

Supplementary Figure 15: Data in support of **Figure 7** and , including analysis of RNA levels and EU incorporation and assessment of KAT5 activity and protein translation rates in primary glioma tumor samples.

Supplementary Figure 16: Data in support of Figures 7 and 8, including analysis of *KAT5*, *MYC*, and *MYCN* expression in GSCs and clinical brain tumor data sets.

Supplementary Figure 17: FACS gating strategies for live, intact, single cells and CD45 detection.

Supplementary Figure 18: FACS gating strategies for AHA, EdU, p27, and histone H4-Ac.

Supplementary Tables
Supplementary Table 1: G0-trap screen results from GSC-0827 cells.

Supplementary Table 2: Gene expression analysis for *in vitro* KAT5 vs CD8 KO in GSC-0827.

Supplementary Table 3: Gene set enrichment analysis for top 200 expressed genes from each cluster for *in vitro* KAT5 vs CD8 KO in GSC-0827, associated with **Figure 2**.

Supplementary Table 4: Gene set enrichment analysis for top 200 depleted genes from each cluster for *in vitro* KAT5 vs CD8 KO in GSC-0827, associated with **Figure 2**.

Supplementary Table 5: GBM gene expression modules used for scRNA-seq data analysis.

Supplementary Table 6: Analysis of KAT5 KO induced changes across 9 GSC isolates, associated with **Figure 3** and **Supplementary Figure 6**.

Supplementary Table 7: Gene expression analysis for GSC-0827 tumor reference clusters, associated with **Figure 4** and **Supplementary Figure 8**.

Supplementary Table 8: Gene set enrichment analysis for top expressed genes for GSC-0827 tumor reference clusters, associated with **Figure 4** and **Supplementary Figure 8.**

Supplementary Table 9: Gene set enrichment analysis for top depleted genes for GSC-0827 tumor reference clusters **Figure 4** and **Supplementary Figure 8.**

Supplementary Table 10: Gene expression analysis for GSC-464T tumor reference clusters, associated with **Figure 4** and **Supplementary Figure 11**.

Supplementary Table 11: KAT5 binding sites in C13 tumors, associated with **Figure 5a**.

Supplementary Table 12: Full Venn diagram gene lists associated with **Figure 5c** and screen results for essential transcription factors in GSC-0827 cells.

Supplementary Table 13: ChromHMM analysis of H3K4me2, H3K27ac, and H3K27me3 marks in Dox+/KAT5+ GSC-0827 Dox-KAT5 tumors, in support of **Figure 5f**.

Supplementary Table 14: Differential Binding (DiffBind) analysis of H3K27ac marks in Dox+ versus Dox- GSC-0827 Dox-KAT5 tumors using DESeq2, in support of **Figure 5**.

Supplementary Table 15: Differential Binding (DiffBind) analysis of H3K27me3 marks in Dox+ versus Dox- GSC-0827 Dox-KAT5 tumors using DESeq2, in support of **Figure 5**.

Supplementary Table 16: Transcription factor binding sites predicted by Homer for genomic regions associated with significantly scoring changes in H3K27ac and H3K27me3 marks from **Supplementary Tables 14-15**, in support of **Supplementary Figure 12**.

Supplementary Table 17: Super enhancers predicted for $KAT5^{on}$ and $KAT5^{off}$ GSC-0827 tumors, in support of **Supplementary Figure 12**.

Supplementary Table 18: Description of glioma tumor samples used for **Fig. 7** and **Supplementary Figure 15**.

Supplementary Table 19: Key Resources

Supplementary Figure 1

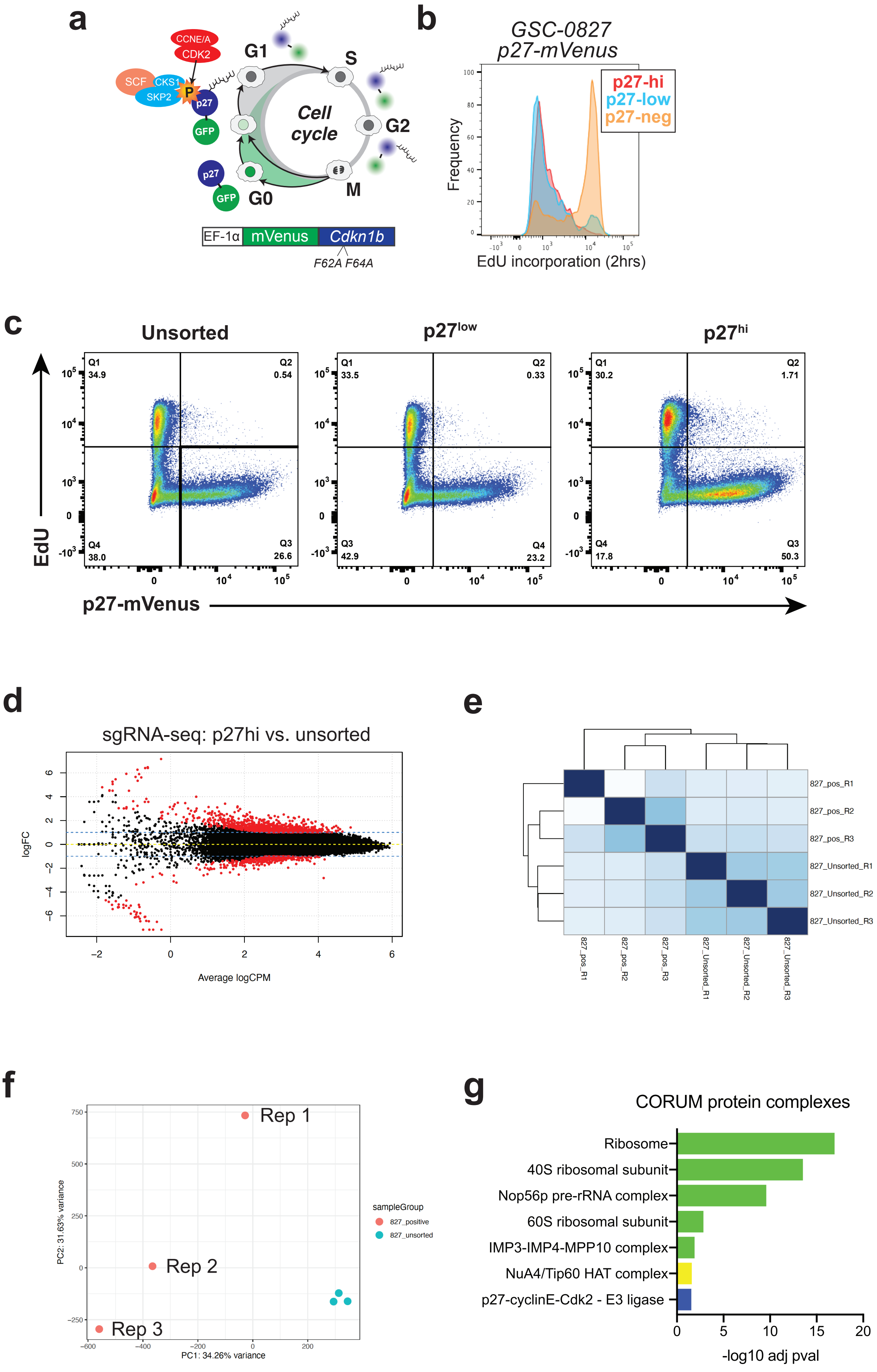

Supplementary Figure 1: Data supporting **Figure 1**.
a-c Validation of G0-reporter in GSC-0827 cells.
a, The p27-mVenus G0/quiescence reporter and its regulation during the cell cycle. For S/G2/M phases of the cell cycle, p27 is targeted for proteolysis by the SCF^{Skp2} E3 ubiquitin ligase complex (Chu et al., 2008). p27 is additionally regulated in G1 via targeted degradation by the Kip1 ubiquitylation-promoting complex at the G0-G1 transition (Chu et al., 2008). As a result, p27 protein only accumulates during G0. The p27 reporter was constructed with a p27 allele harboring two amino acid substitutions (F62A and F64A) that block binding to Cyclin/CDK complexes (preventing functional activity) but do not interfere with its cell cycle-dependent proteolysis (Oki et al., 2014).
b, Flow-based examination of EdU incorporation (2hrs) of p27 high, low, and negative GSC-0827 cells using the p27-mVenus reporter. Consistent with reporting of G0-like states in GSCs, EdU incorporation is significantly suppressed in p27-mVenus+ and especially p27-mVenus-hi cells. p27-mVenus-hi cells= top 20%. p27-mVenus-low cells = bottom 20%. Results were replicated in an additional independent experiment.
c, DNA replication and p27 assay for outgrown populations of p27-hi sorted GSC-0827s. Controls include p27-low sorted and non-sorted cells. To ensure that p27-hi cells could re-enter the cell cycle, p27-hi cells were sorted, recultured, and assayed 7 days later for EdU incorporation and p27-mVenus levels. The p27-hi cells showed high, but somewhat diminished, EdU incorporation rate of 30% versus 35% for control cells, and also had higher residual p27-mVenus expression. These results are consistent with a preponderance of p27-hi cells being division capable and entering the cell cycle with delayed and somewhat variable kinetics as cells exit from G0. Results were replicated in an additional independent experiment.
d-g, Supporting data for G0-trap screen in **Figure 1** (n= 3 screen replicates).
d, M (log fold change) versus A (mean expression) plot from sgRNA-seq for G0-trap screen. Red indicates positively scoring enriched or depleted sgRNA.
e, Correlation plot of screen replicates.
f, Principal component analysis of screen replicates.
g, Gene set enrichment analysis of screen hits using CORUM protein complex database (http://mips.helmholtz-muenchen.de/corum/).

Supplementary Figure 2

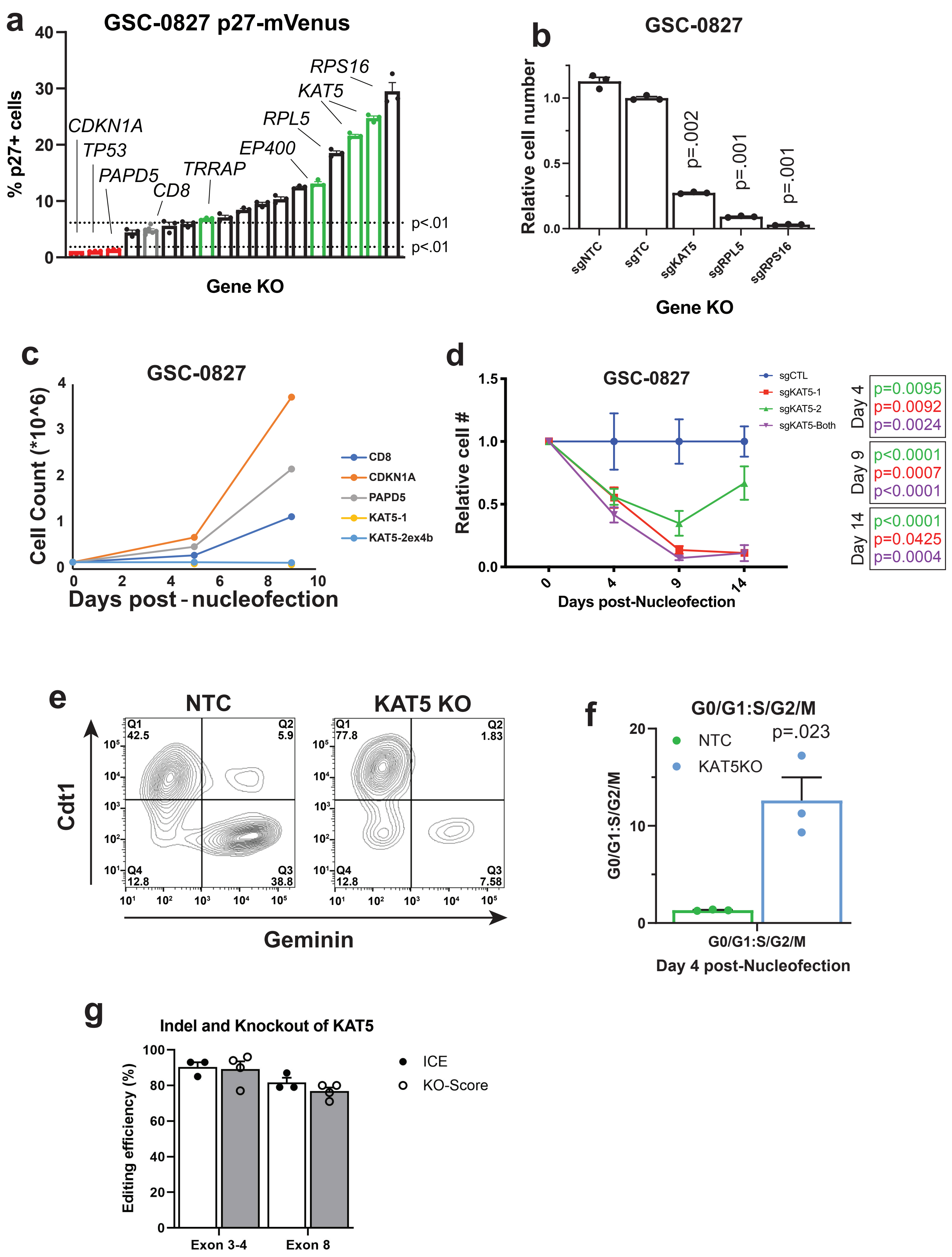

Supplementary Figure 2: KAT5 inhibition triggers G0-like state in GSCs in *vitro*, in support of **Figure 1**.

a, Select retest of G0-trap screen hits using nucleofection of sgRNA:Cas9 RNPs in GSC-0827 p27-mVenus cells. Cells were flow analyzed 5 days post-nucleofection (n=3; unpaired, two tailed student's t-test). This technique allows for penetrant KO of target genes in GSCs in a relatively short period time (~48hrs) (Hoellerbauer et al., 2020a).

b, Comparison cell number in GSC-0827 cells after KAT5, RPL5, and RPS16 inhibition (n=3; unpaired, two tailed student's t-test).

c, Cell counts at various times post-nucleofection of KAT5 and two negative screen hits found significantly depleted in GSC-0827 $p27^{hi}$ cells, CDKN1A and PAPD5. Experiment was performed twice.

d, Outgrowth of GSC-0827 cells after nucleofection of sgKAT5:Cas9 or sgCTRL:Cas9 RNP complexes (n=3; unpaired, two tailed student's t-test).

e, Flow analysis of FUCCI factors in GSC-0827 cells 5 days post-nucleofection of sgKAT5:Cas9 or sgCTRL:Cas9 RNP complexes.

f, Quantification of G0/G1:G2/M ratios from **e** (n=3; unpaired, two tailed student's t-test).

g, Analysis of indel efficiency and knockout of KAT5 using sgRNA:Cas9 RNPs in GSC-0827 cells (n=3).

Supplementary Figure 3

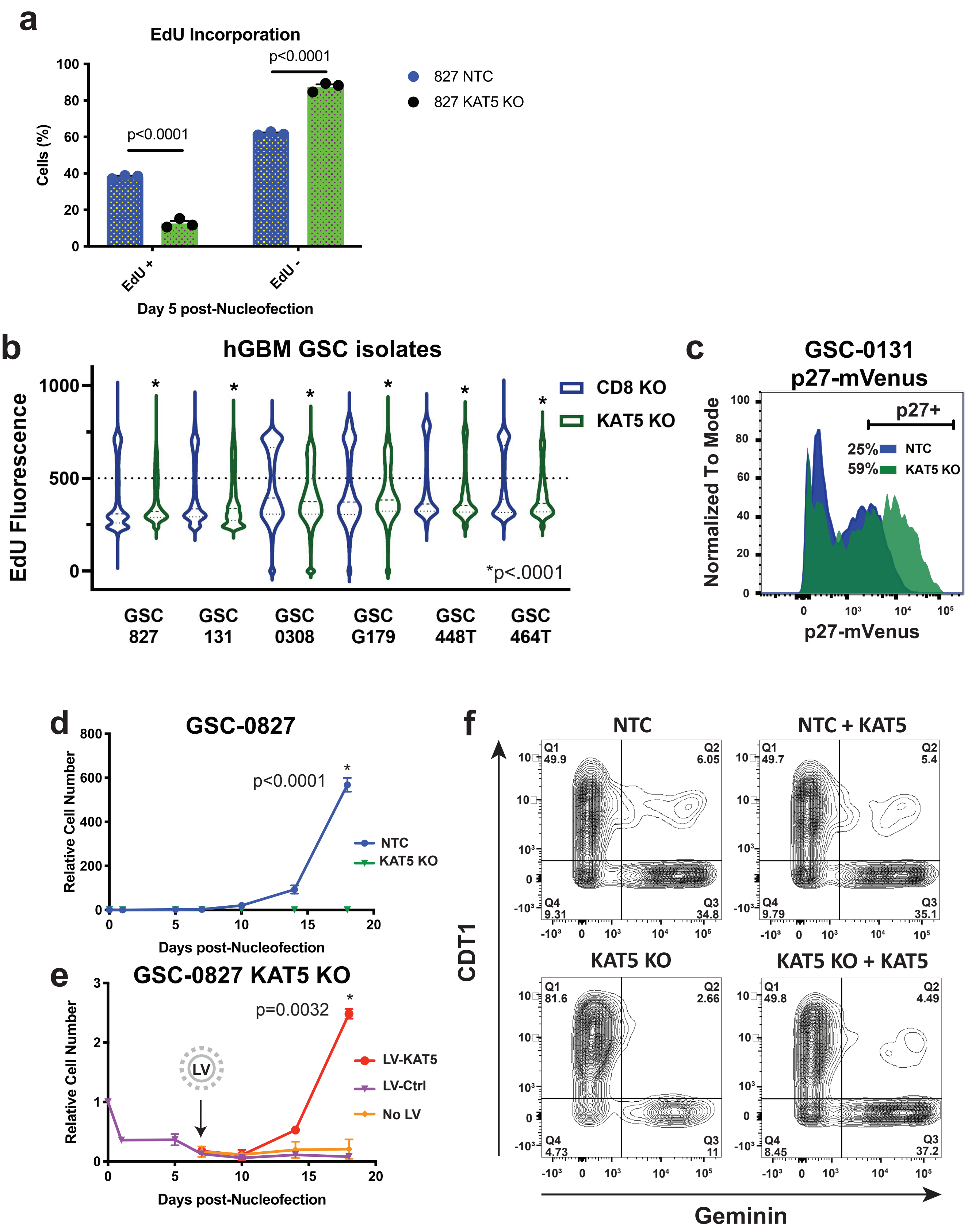

Supplementary Figure 3: *KAT5* inhibition triggers G0-like state in GSCs in *vitro*, in support of **Figure 1**.
a, Quantification of loss of EdU incorporation for this experiment (n=3; student's t-test, p<.01) from **Figure 1e**.
b, Violin plots of 3hrs of EdU incorporation *KAT5* KO 5 days post-nucleofection in various GSC isolates. KS test was used to test significance (p<.0001; n>5489 cells).
c, Retest of *KAT5* inhibition in GSC-0131 p27-mVenus cells using nucleofection of sgRNA:Cas9 RNPs. Cells were flow analyzed 5 days post-nucleofection.
d, Evaluation of growth of *KAT5* KO GSC-0827 cells at days shown post-nucleofection. (n=3; student's t-test, p<.001).
e, *KAT5* KO rescue and reversibility experiment. To examine reversibility of KAT5 KO growth defect, we first knocked out *KAT5* using sgKAT5:Cas9 in GSC-0827 cells and then added *KAT5* back 7 days later via lentiviral transduction as an expressed KAT5 ORF. By 7 days, the cells that had received the LV-KAT5 ORF had growth rates return to near parental cell levels while control LV cells were no different than KAT5 KO in **d**.
f, Flow analysis of FUCCI factors after rescue 14 days post-nucleofection and 10 days post-rescue. After 10 days, the LV-*KAT5* ORF cells had their cell cycle phase ratios return to parental cell levels, as judged by FUCCI reporter analysis, while the LV-control cells remained similar to *KAT5* KO.

Supplementary Figure 4

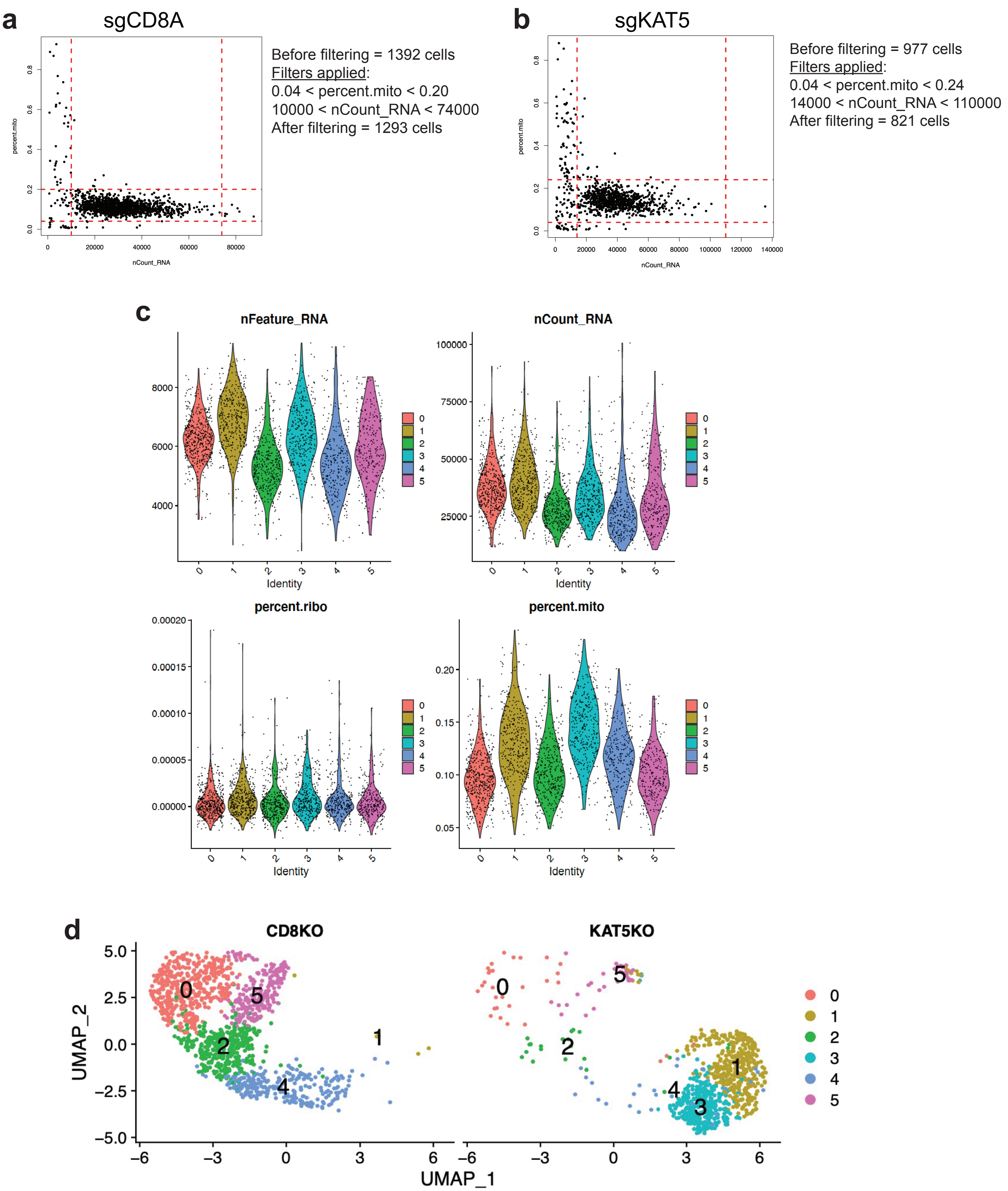

Supplementary Figure 4: scRNA-seq filtering and data quality evaluation for **Figure 2**.
a-b, Filtering criteria used for sgCD8A and sgKAT5 GSC-0827 scRNA-seq analysis.
c, Visualization of scRNA-seq quality control parameters for co-embedded KAT5 and CD8 KO GSC-0827 scRNA-seq data showing cluster scheme from **Figure 2**.
d, Individual UMAP projections of the co-embedded CD8A and KAT5 KO GSC-0827 scRNA-seq data from **Figure 2**.

Supplementary Figure 5

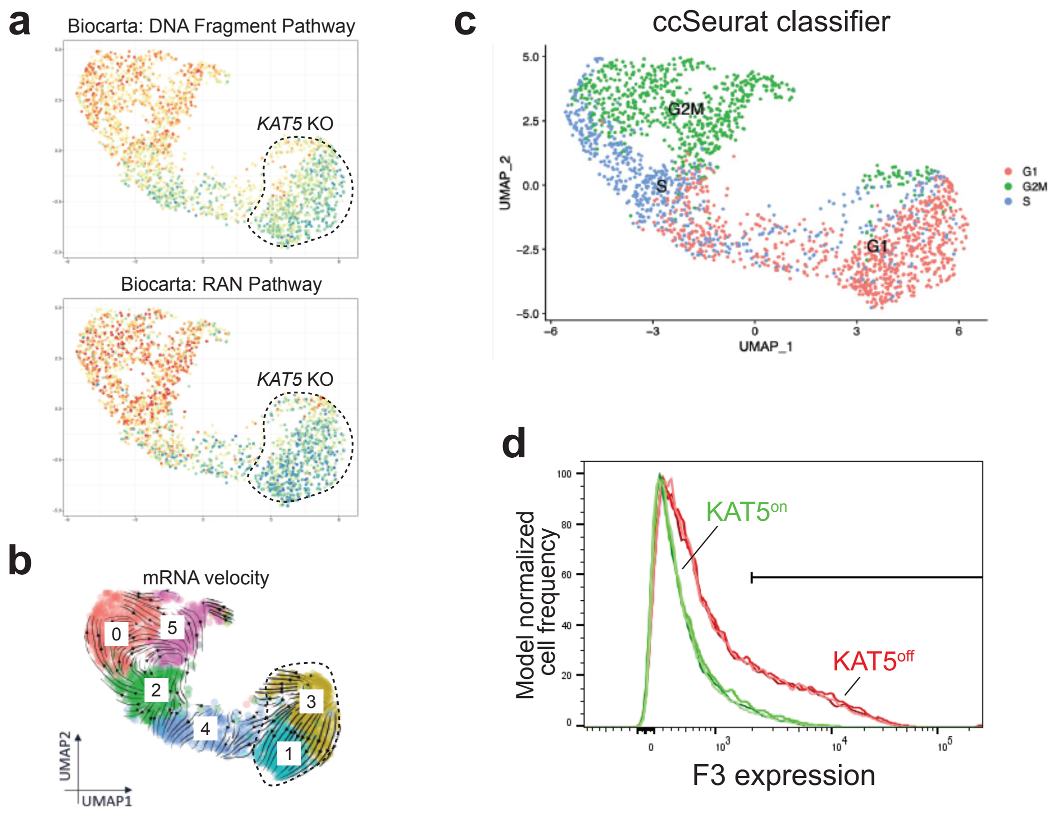

Supplementary Figure 5: Single cell gene expression analysis of KAT5 KO in GSC-0827 cells *in vitro*, in support of **Figure 2**.
a, Gene Set Variation Analysis (GSVA) associated with clusters and cell cycle phases showing MCM pathway required for initiation of DNA replication in early S-phase.
b, RNA velocity analysis of scRNA-seq data from **Figures 2a-d**.
c, Cell cycle state predictions using the ccSeurat classifier for scRNA-seq data from Figure 2**a**.
d, F3 expression evaluated by flow analysis of GSC-0827 C13 cells grown in the presence of Dox ($KAT5^{on}$) or 5 days post withdrawal ($KAT5^{off}$) (n=3).

Supplementary Figure 6

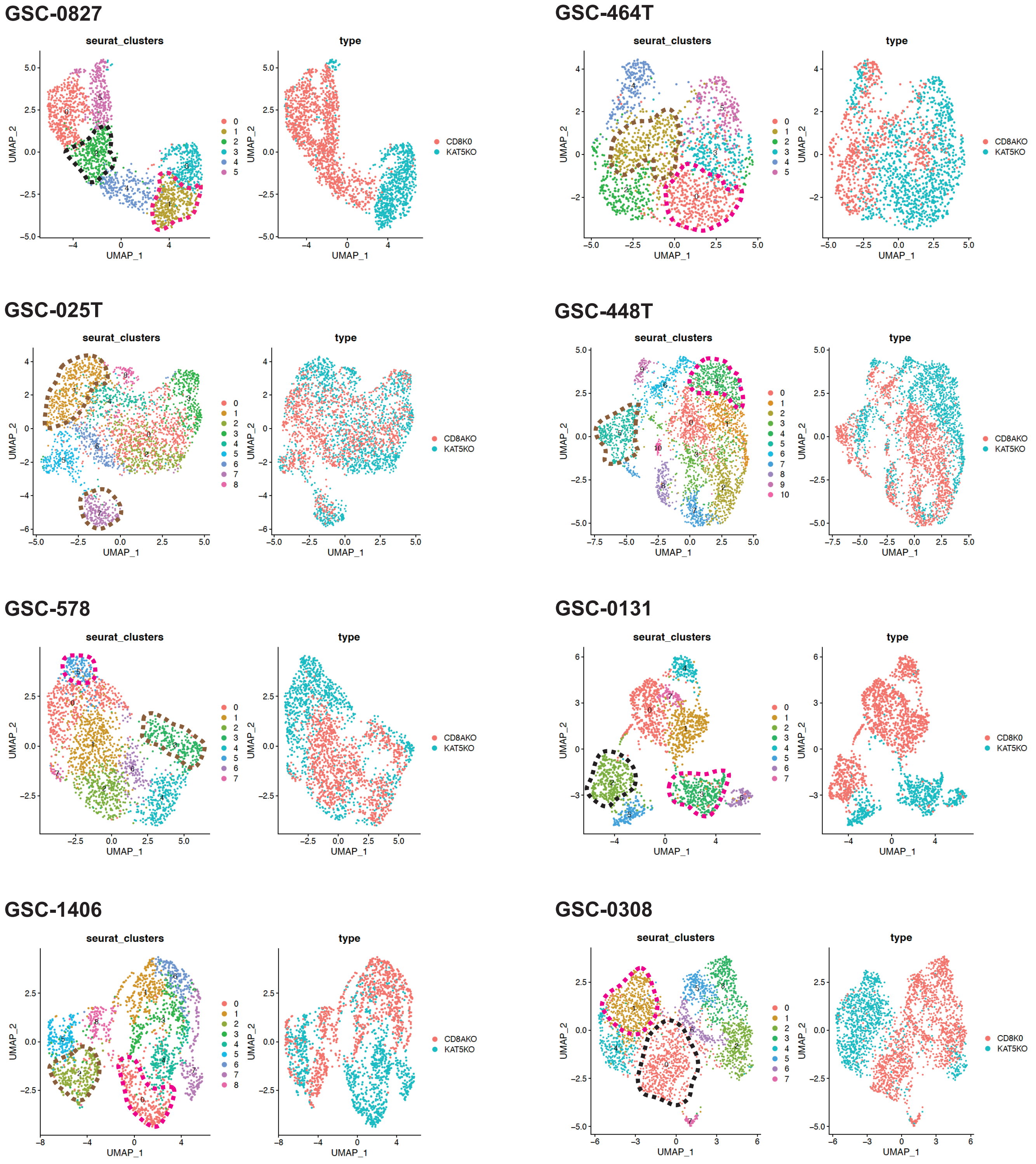

Examples of:

= Control clusters (mainly CD8KO cells)

= Novel clusters (mainly KAT5KO cells)

= Mixed clusters

Note: each cluster is designated as one of these categories for gene expression analysis. Two examples of clusters were called out at random for each data set above. Some GSCs are mainly composed of solely of Control and Novel clusters, others contain Mixed clusters as well.

Supplementary Figure 6: Single cell RNA-seq data analysis associated with **Figure 3**. 9 different GBM GSC isolates were used to examine changes in gene expression states in *KAT5* KO and control *CD8* KO as detailed in **Figure 3**. For each isolate, we co-embedded scRNA-seq data from *KAT5* KO and control *CD8* KO cells into a single merged scRNA-seq object. Then, *de novo* clustering was applied to each GSC line, and each cluster was examined for admixtures of *KAT5* and *CD8* KO cells.

Supplementary Figure 7

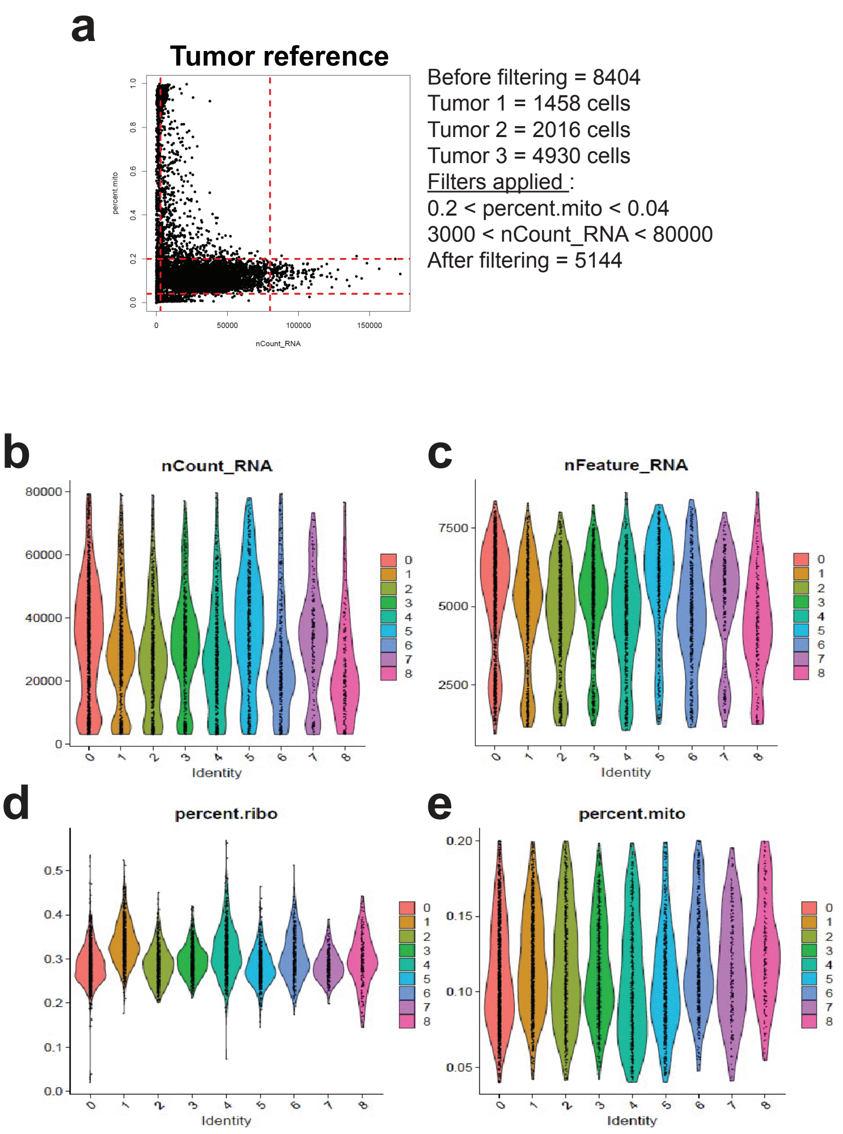

Supplementary Figure 7: scRNA-seq filtering and data quality evaluation for **Figure 4**.
a, Experimental overview for collection of samples for scRNA-seq.
a, Filtering criteria used for each scRNA-seq data set.
b-e, scRNA-seq quality control metrics viewed by cluster after filtering of data.

Supplementary Figure 8

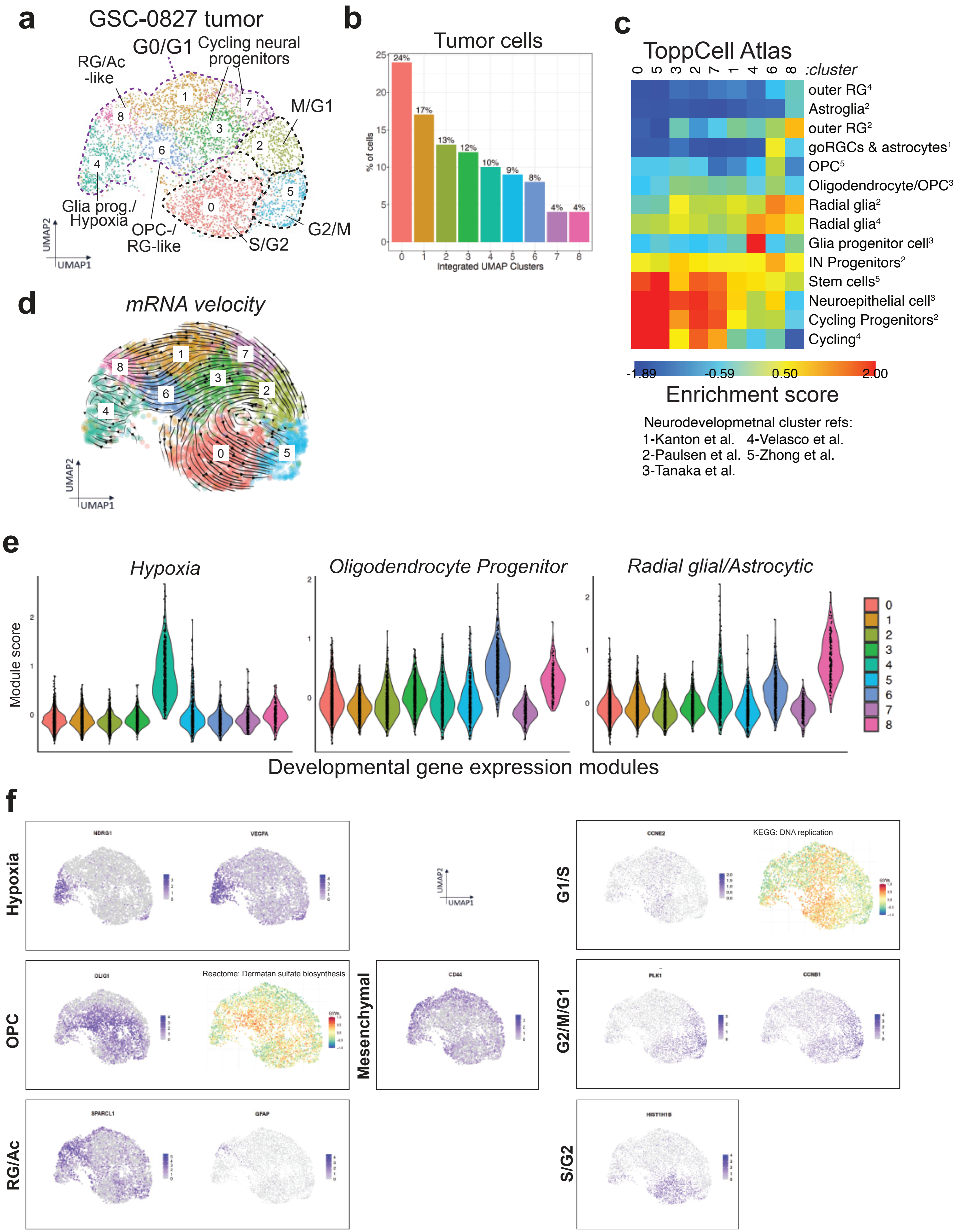

Supplementary Figure 8: Data associated with **Figure 4**.
a, Projections of scRNA-seq data for GSC-0827 tumor reference using 3 tumor samples, along with inferred cluster cell cycle phase and neurodevelopmental associated cell type. Quality control metrics are shown in **Supplementary Fig. 7** and were used for downstream normalization and dimensional reduction using principal component analysis (PCA) and subsequent shared nearest neighbors clustering (Satija et al., 2015). Data was visualized using uniform manifold approximation and projection (UMAP) for dimensional reduction of data and generation of *de novo* cell-based clusters (Becht et al., 2018). Each data point = single cell. Supporting data includes: top enriched genes for each cluster (**Supplementary Data 7**); gene set enrichment (**Supplementary Data 8 & 9**); gene expression modules (**Supplementary Data 5**); and individual and gene set expression profiles (**Supplementary Figs. 9 & 10**).
b, Proportion of cells in each cluster from **a**.
c, Heatmap showing enrichment scores for scRNA-seq clusters in GSC-0827 tumor reference (A) for select neurodevelopmental cell types found in the ToppCell Atlas. A complete version of this figure is available in **Supplementary Fig. 10**.
d, RNA velocity analysis of scRNA-seq data for **a** using scVelo (https://scvelo.readthedocs.io/) (Bergen et al., 2020).
e, Select gene expression module scores for clusters in in GSC-0827 tumor reference. A list of genes in each module can be found in **Supplementary Data 5**.
f, Gene expression analysis of individual genes and Gene Set Variation Analysis (GSVA) associated with clusters and cell cycle phases for GSC-0827 tumor reference associated with **a**. Note that Reactome: Dermatan sulfate biosynthesis includes category includes BCAN, CSPG4, CSPG5, NCAN, and VCAN, which are all enriched in cluster 6 (OPC).

Supplementary Figure 9

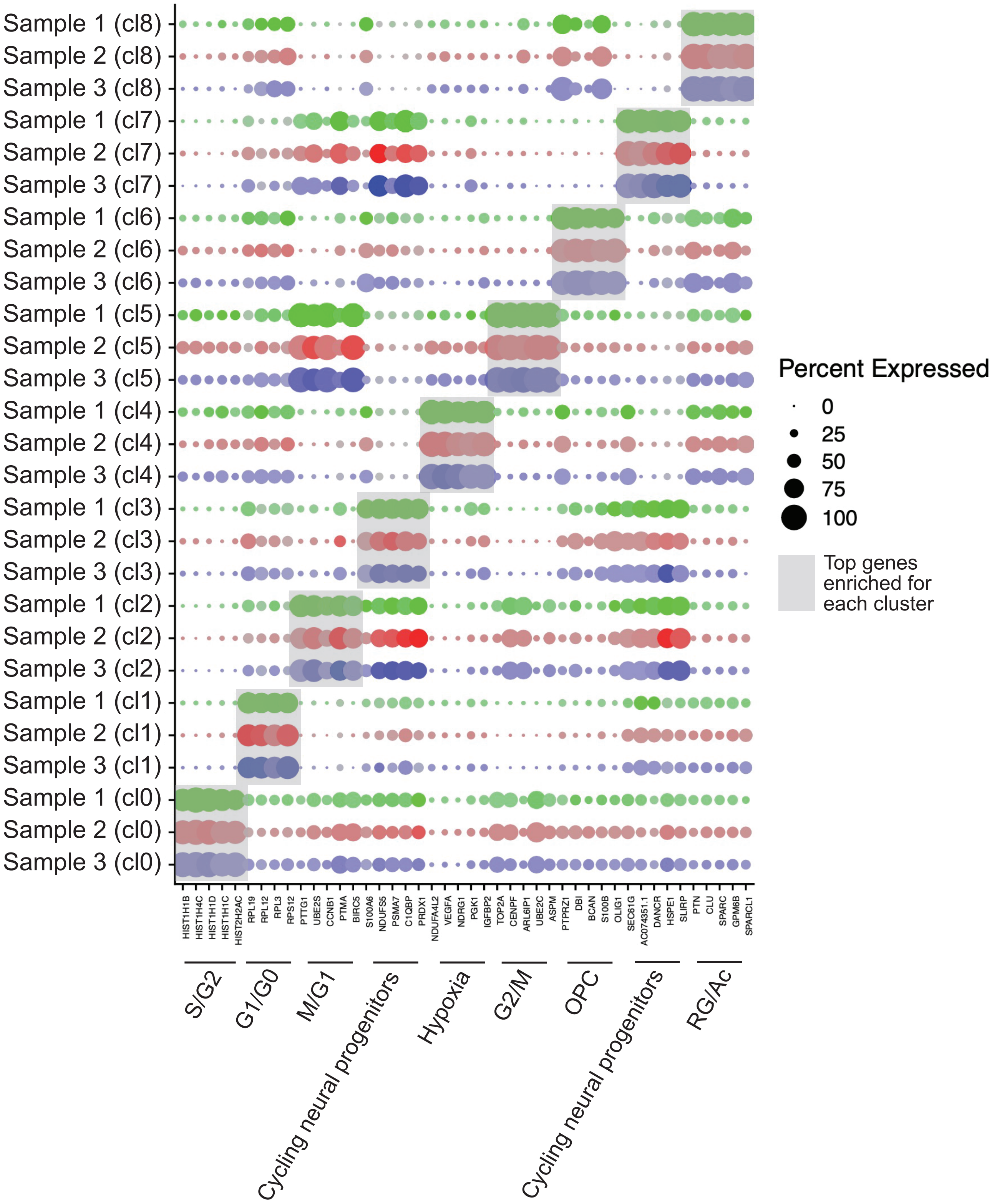

Supplementary Figure 9: Dot plot top differentially expressed genes in UMAP clusters for GSC-0827 tumor scRNA-seq data, associated with **Figure 4** and **Supplementary Figure 8**. The data is derived from 3 scRNA-seq data sets from GSC-0827 tumors (i.e., samples 1-3). The samples are associated by cluster (cl) from cl0 to cl8. Genes were ranked based on adjusted p-values from differentiation expression analysis of for each cluster **Supplementary Data 7**). The size of the dots corresponds to the proportion of cells within the cluster expressing the gene (% Exp.). Cluster designations from **Supplementary Fig. 8a** are shown below each cluster-specific gene set.

Supplementary Figure 10

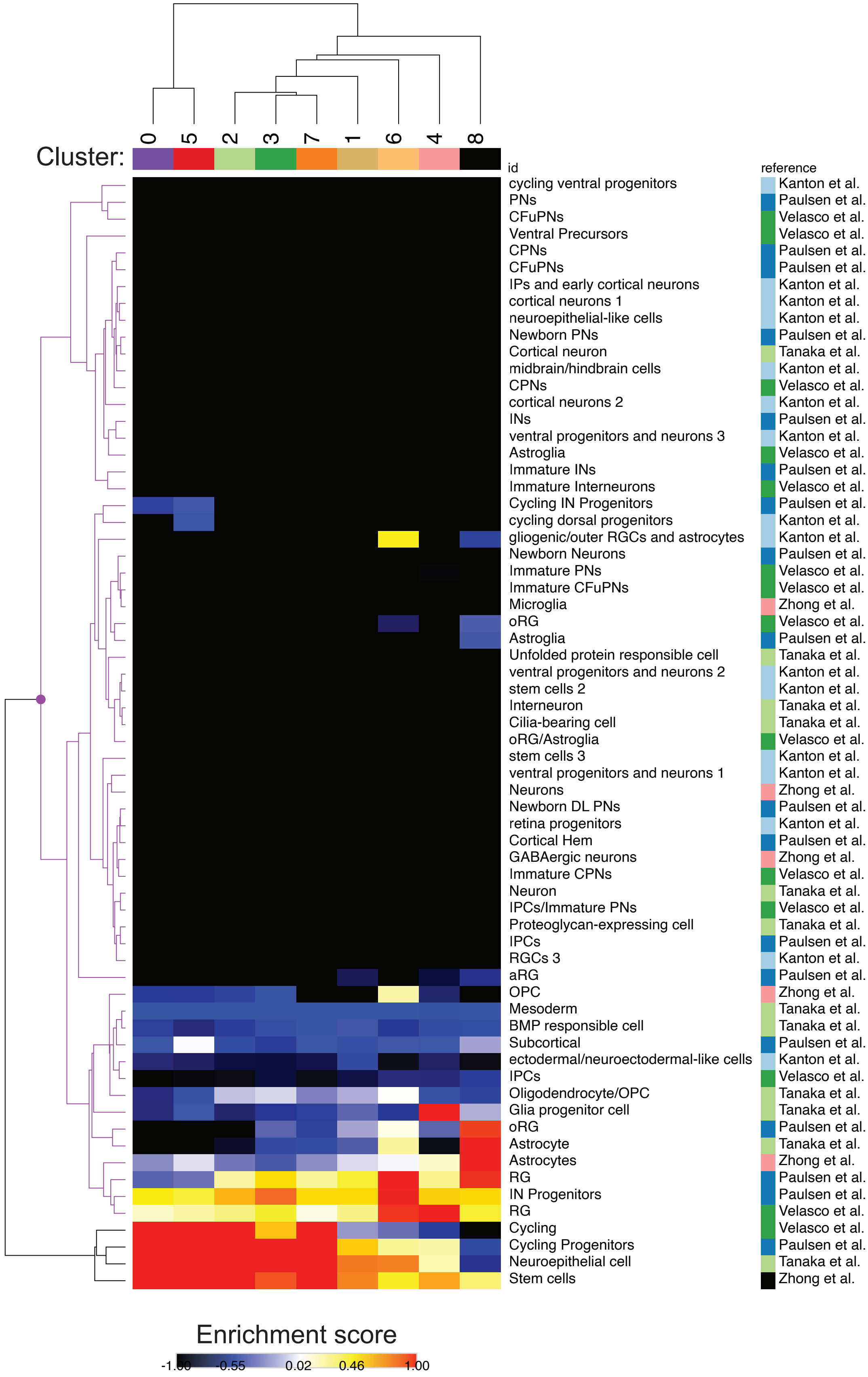

Supplementary Figure 10: The full heatmap of neurodevelopmental gene expression profiles from **Supplementary Fig. 8c** for human fetal and adult neurodevelopmental cell types in the scRNA-seq ToppCell Atlas. Tumor cluster gene sets enriched in tumor clusters to those for neurodevelopmental cell types available in the ToppCell Atlas (Jin et al., 2021) using linear regression analysis (Young et al., 2018). ToppCell Atlas data and cell descriptions are taken from four references shown on the right-side column (PMID: 31619793; PMID: 32049002; PMID: 31168097; PMID: 29539641).

Supplementary Figure 11

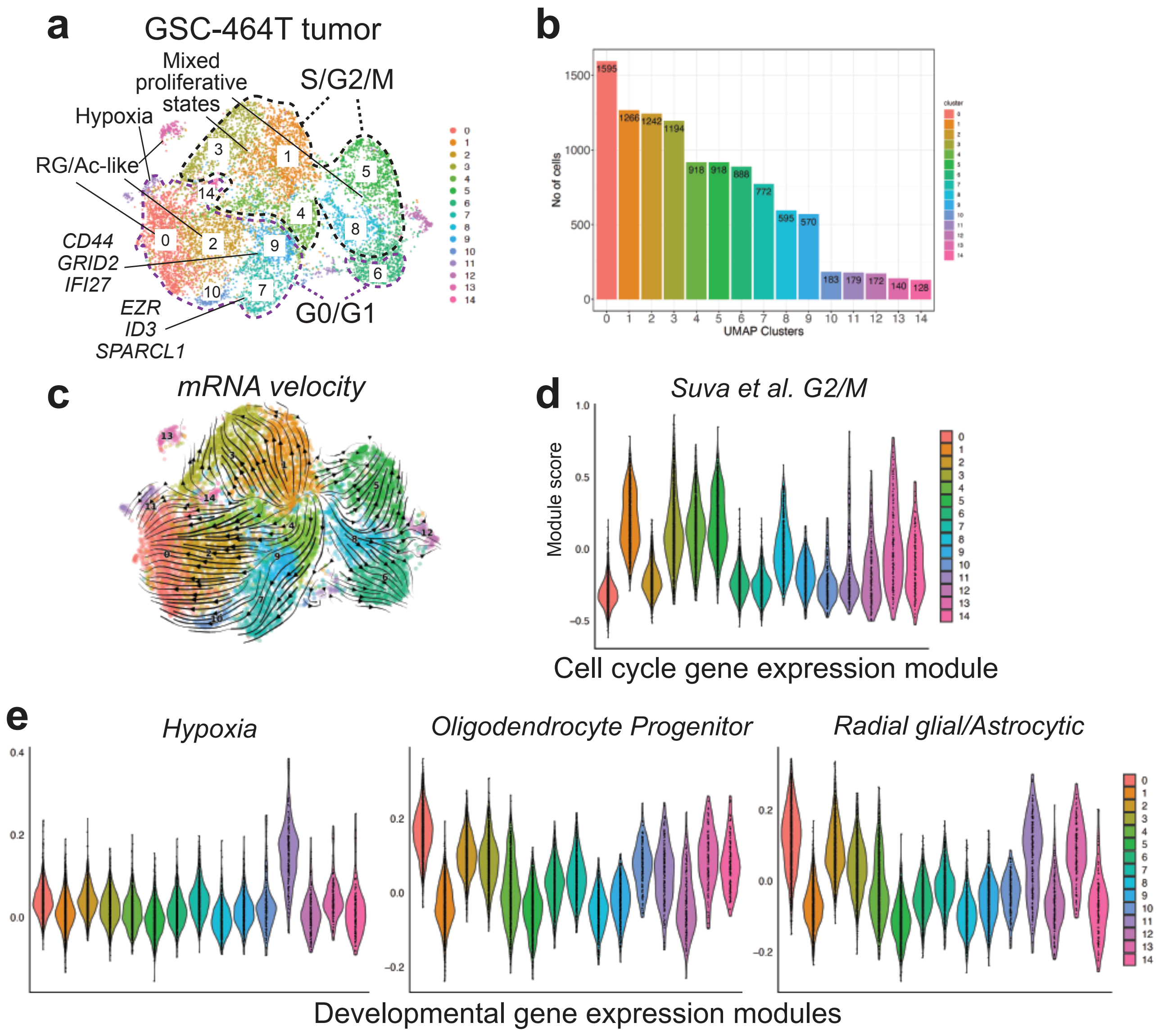

Supplementary Figure 11: Data associated with **Figure 4**.
a, Projections of scRNA-seq data for GSC-464T tumor reference, along with inferred cluster cell cycle phase and neurodevelopmental associated cell type. Supporting data includes: top enriched genes for each cluster (**Supplementary Data 10**);
b, Proportion of cells in each cluster from **a**.
c, RNA velocity analysis of scRNA-seq data for a using scVelo (https://scvelo.readthedocs.io/) (Bergen et al., 2020).
d, e, Select gene expression module scores for clusters in GSC-464T tumor reference. A list of genes in each module can be found in **Supplementary Data 5**.

Supplementary Figure 12

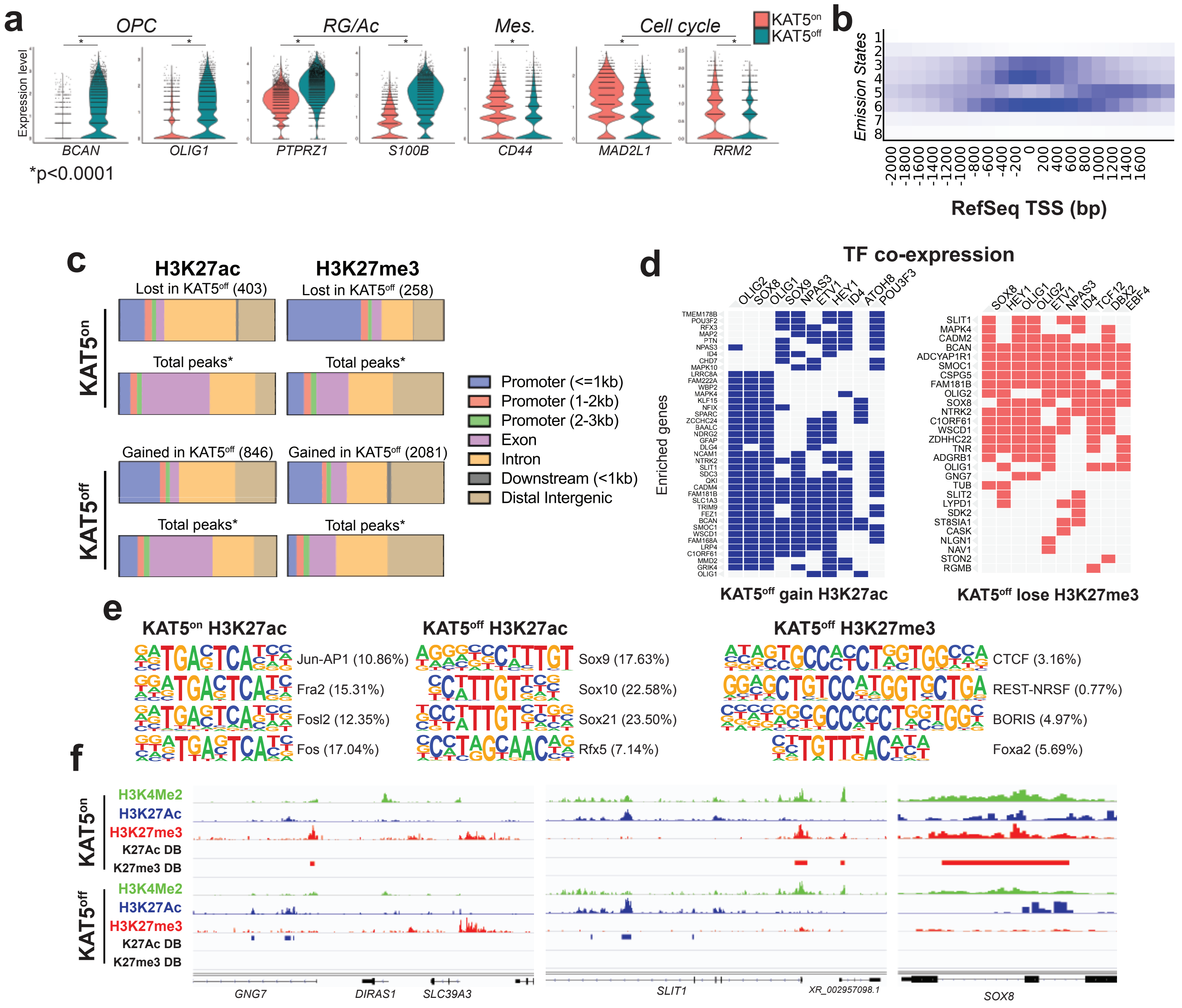

g KAT5on Superenhancers

Chrom.	Start	End	Width	Slope	Gene	Description	Relevance
chr7	13032102	13063402	31301	1563.6	***ARL4A***	ADP ribosylation factor like GTPase 4A	glioma-associated antigen
chr7	54813258	54813492	235	627.5	***SEC61G****	SEC61 translocon subunit gamma	GBM: survival / ER stress
chr7	101154436	101167265	12830	489.1	***VGF***	VGF nerve growth factor inducible	GBM: stemness / survival
chr6	150753222	150775852	22631	189.8	***MTHFD1L***	methylenetetrahydrofolate dehydrogenase 1 like	folate enzyme / oncogenesis
chr1	164576043	164612598	36556	82.4	***PBX1***	PBX homeobox 1	SVZ stem and progenitor cells
chr19	44742168	44753542	11375	72.0	***BCL3***	BCL3 transcription coactivator	GBM: EMT/ NF-kB
chr1	183000000	183000000	50644	48.3	***RGS16***	regulator of G protein signaling 16	GBM: mesenchymal
chr7	92755155	92769276	14122	48.2	***CDK6***	cyclin dependent kinase 6	GBM: G1/S transition
chr11	35151288	35166564	15277	25.5	***CD44***	CD44 cell-surface glycoprotein	GBM: mesenchymal

**adjacent to EGFR and part or EGFR amplicon in GSC-0827 cells.*

h KAT5off Superenhancers

Chrom.	Start	End	Width	Slope	Gene	Description	Relevance
chr1	230728655	230747162	18508	18507.0	***AGT***	angiotensinogen	GBM: bevacizumab biomarker
chr1	156406287	156421983	15697	15696.0	***C1ORF61***	MIR9-1 host gene	Brain development / oncogenesis
chr17	44907324	44917306	9983	9982.0	***GFAP***	glial fibrillary acidic protein	Astrocytes and astryocytoma
chr19	2685571	2696991	11421	552.3	***GNG7***	G protein subunit gamma 7	GBM: expression
chr3	47536092	47554182	18091	104.8	***CSPG5***	chondroitin sulfate proteoglycan 5	OPC progenitors
chr2	119370840	119387743	16904	23.1	***DBI***	diazepam binding inhibitor, acyl-CoA binding protein	quiescent cancer cells
chr1	156638190	156655316	17127	15.9	***BCAN***	brevican	GBM: expression / invasiveness
chr14	69963927	69964272	346	9.2	***SMOC1***	SPARC-related modular calcium-binding protein 1	Glioma/GBM/OPC: expression
chr16	986231	991762	5532	2.1	***SOX8***	SRY-box transcription factor 8	OLIG1+ low grade glioma

Supplementary Figure 12: Data in support of **Figure 5**, including scRNA-seq analysis and CUT&TAG analysis of GSC-0827 Clone 13 tumors.

a, Violin plots of expression values for genes associated with gene expression modules from **Figure 5e** from scRNA-seq data for $KAT5^{on}$ and $KAT5^{off}$ C13 tumors. Each data point = single cell. Each data point = single cell. KS test was used to test significance (p<.0001)(4951 Dox+ cells; 5507 Dox- cells).

b, ChromHMM analysis of genomic regions around transcription start sites in $KAT5^{on}$ C13 tumor cells showing 8 possible chromatin states (i.e., emission states) for H3K4me2, H3K27ac, and H3K27me3. The darker blue color corresponds to a greater probability of observing the mark in the state. The full data set is available in **Supplementary Data 11**.

c, Comparison of distribution of H3K27ac and H3K27me3 peaks from tumors among genomic regions for peaks significantly different in $KAT5^{on}$ and $KAT5^{off}$ conditions. Total significant peaks shown in parentheses; *MACS2 called peaks >11,000 for ac and >47,000 for me3.

d, Gene-based output from **c** using colored squares indicate genes enriched in specific TF associated gene sets from ARCHS4 database from **Figure 5h**.

e, Enrichment for known transcription factor binding sites associated with H3K27ac and H3K27me3 sites are enriched in $KAT5^{on}$ and $KAT5^{off}$ C13 tumors. Dox+ condition for H3K27me3 did not yield significant enrichments. The complete datasets can be found in **Supplementary Data 16**.

f, Additional examples of genes with significantly altered H3K27ac/me3 peaks in $KAT5^{on}$ and $KAT5^{off}$ C13 tumors.

g, Call outs of super enhancers among 521 predicted from $KAT5^{on}$ GSC-0827 tumors.

h, Call outs of super enhancers among 218 predicted from $KAT5^{off}$ GSC-0827 tumors. Complete data sets can be found in **Supplementary Data 17**.

Supplementary Figure 13

a

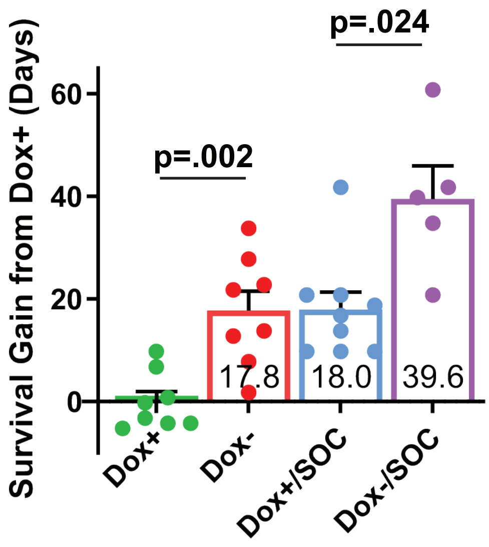

b

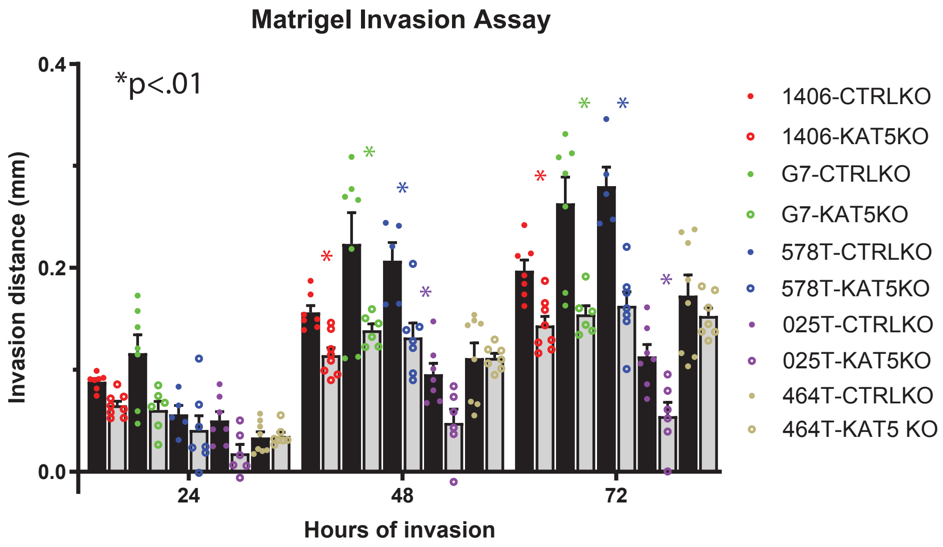

Supplementary Figure 13: Data in support for **Figure 6**.
a, Survival gain is measured as additional days compared to Dox+ control. SOC treatment in the absence of KAT5 (Dox-/SOC) prolongs survival with 21.6 additional days compared to when KAT5 is present (Dox+/SOC) (unpaired, two tailed student t-tests, p=.006). Other t-tests with significance include Dox+ vs. Dox- (p=.0009), Dox+ vs. Dox+/SOC (p=.0004), Dox+ vs Dox-/SOC (p=1.9E-05), and Dox- vs Dox-/SOC (p=.009).
b, Invasion assays for 5 additional GSC isolates with and without *KAT5* KO. 5 days post *KAT5* KO cells were grown as spheres for 3 days and transferred to Matrigel covered wells for the specified number of hours. Phase-contrast images were captured every 24 hours for 72 hours. The area covered by invading cells was measured using FIJI. (significance was tested using repeated measurements 2-way ANOVA with Giesser-Greenhouse correction and Sidak multiple corrections test; n ≥ 5; $p_{1406(24hr)}=0.001$, $p_{1406(48hr)}=0.02$, $p_{1406(72hr)}=0.005$, $p_{G7(72hr)}=0.01$, $p_{578T(48hr)}=0.003$, $p_{578T(72hr)}<0.0001$, $p_{025T(72hr)}=0.02$). Data is in support of **Figure 6j**.
Source data with exact p values are provided with this paper as a Source Data file

Supplementary Figure 14

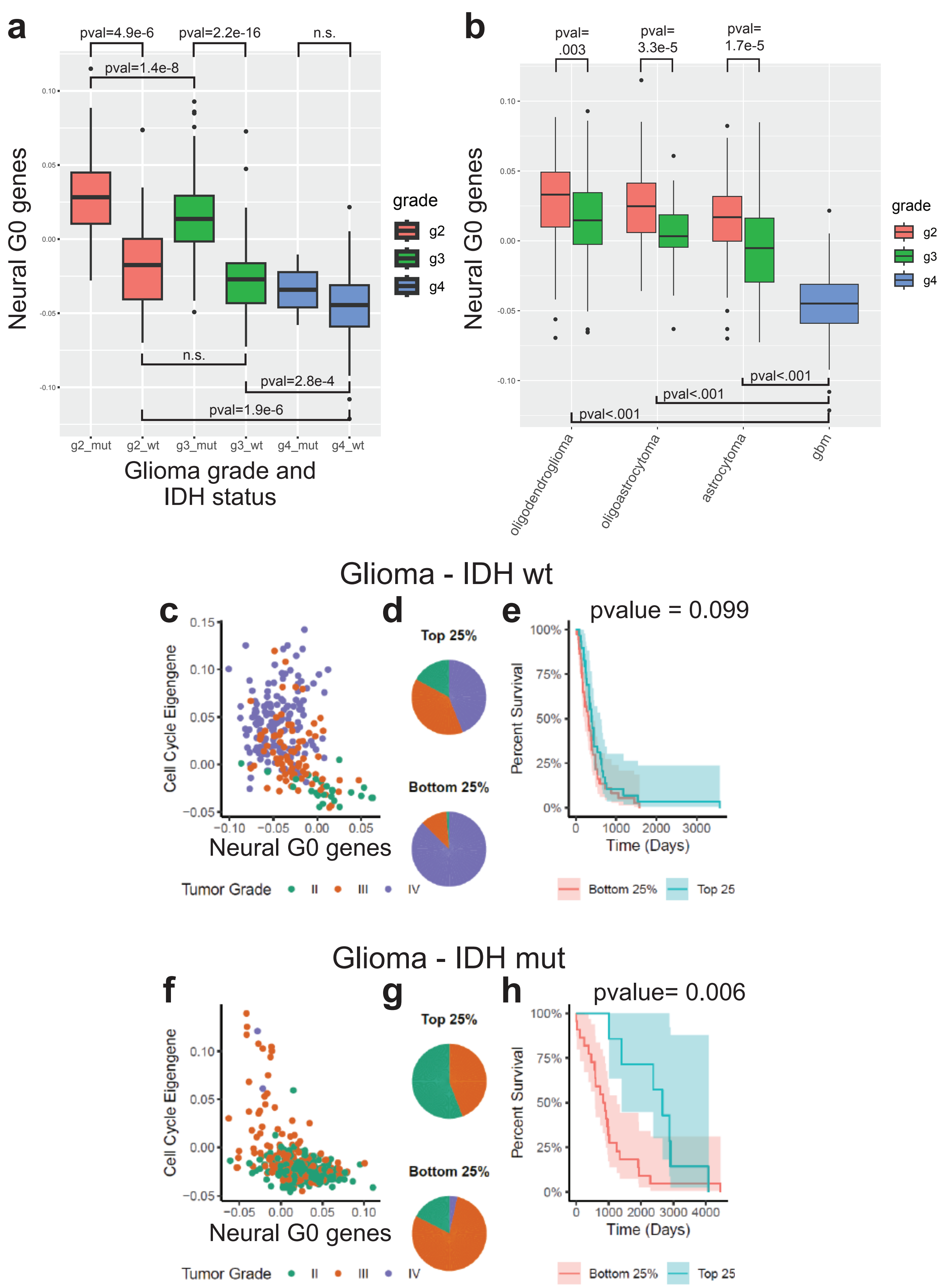

Supplementary Figure 14: Data in support for **Figures 6** and **7**.
a, Relative Neural G0 eigengene expression between grade II (n = 226), III (n = 244), and IV (n = 150) for IDH1/2 wildtype and mutant gliomas (TCGA). All pairwise Student's t-tests had significant p-values.
b, Relative Neural G0 eigengene expression between grade 2 and grade 3 tumors for astrocytomas, oligoastrocytomas, and oligodendrogliomas and also for GBM tumors from (**a**).
c, Comparison of cell cycle and Neural G0 eigengene relative expression in IDH1/2 wt gliomas (TCGA). Each tumor is colored by its grade (green = II, red = III, and purple = IV). This eigengene represents the common variation across each patient tumor for the Neural G0 genes upregulated after loss of *KAT5* activity in tumors, i.e., first principal component corrected for direction if necessary.
d, Distribution of tumor grade between tumors with top 25% and bottom 25% of KAT5-Neural G0 eigengene expression.
e, Kaplan–Meier survival plot of tumors with top 25% and bottom 25% of Neural G0 eigengene expression of Neural G0 genes. A Cox Proportional Hazards regression p-value for survival was used to gauge significance. Shaded region is the 95% confidence interval for the survival curve.
f, **g**, **h**, same as **c-e** except IDH1/2 mut gliomas (TCGA) were used. Note: only 2 IDH1/2 mut grade IV gliomas are available in this data set.

Supplementary Figure 15

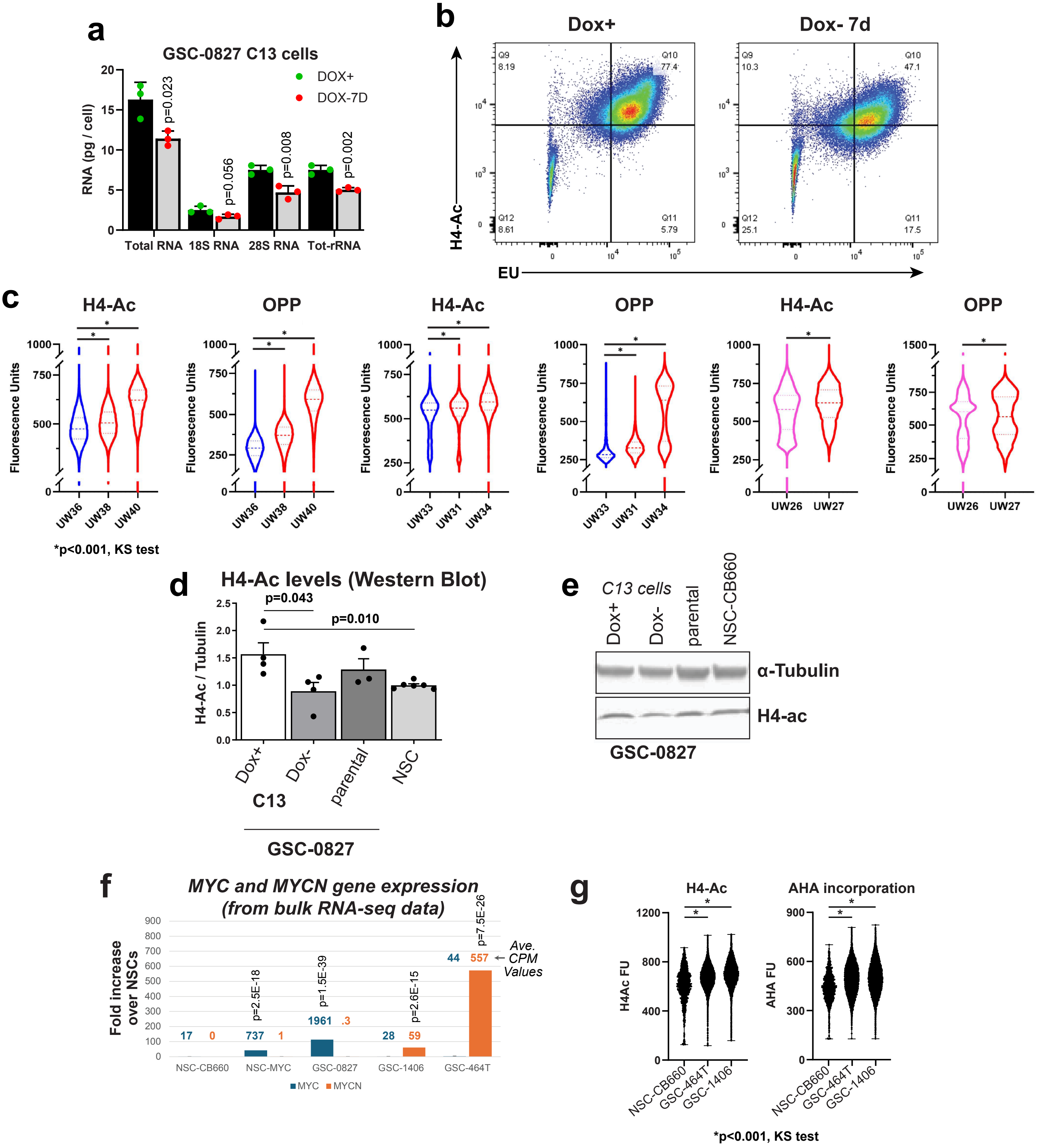

Supplementary Figure 15: Data in support of **Figure 7**, including analysis of RNA levels and EU incorporation and assessment of KAT5 activity and protein translation rates in primary glioma tumor samples.

a, Total RNA and rRNA quantification using TapeStation RNA assay system (Agilent) using RNA isolated from GSC-0827 C13 cells grown in Dox+ and Dox- conditions (7 days).

b, FACS analysis of histone H4 acetylation and EU levels after 0 and 7 days of Dox withdrawal in C13 cells. EU treatment was performed for 30mins before harvest.

c, Violin plots of OPP and pan-H4-Ac assay results for 2 LGG (IDH1/2mut) (blue), 5 HGG (IDH1/2wt) (red), and 1 HGG (IDH1/2 mut) (pink) tumors. KS test was used to assess significance ($p<0.0001$) ($n \geq 2750$ cells/sample).

d, Quantification of Western blot results shown in **e** for H4-Ac (pan) for GSC-0827 and NSC-CB660 cells. C13 = clone 13 Dox-KAT5 cells (Dox+ indicates continuous Dox+ growth; Dox- indicates doxycycline removal for 7 days) (n = 3-6 as shown).

f, MYC and MYCN gene expression data from RNA-seq data for NSCs and GSCs indicated. Significance was determined by comparing replicates of RNA-seq data to NSC-CB660 using DESeq2 (n=3). NSC-CB660-Myc was previously published in Hubert et al., 2013).

g, KAT5 activity (histone H4-Ac) levels and AHA incorporation (protein synthesis) in NSC-CB660, GSC-464T, and GSC-1406 cells ($n \geq 5959$ cells/sample).

Supplementary Figure 16

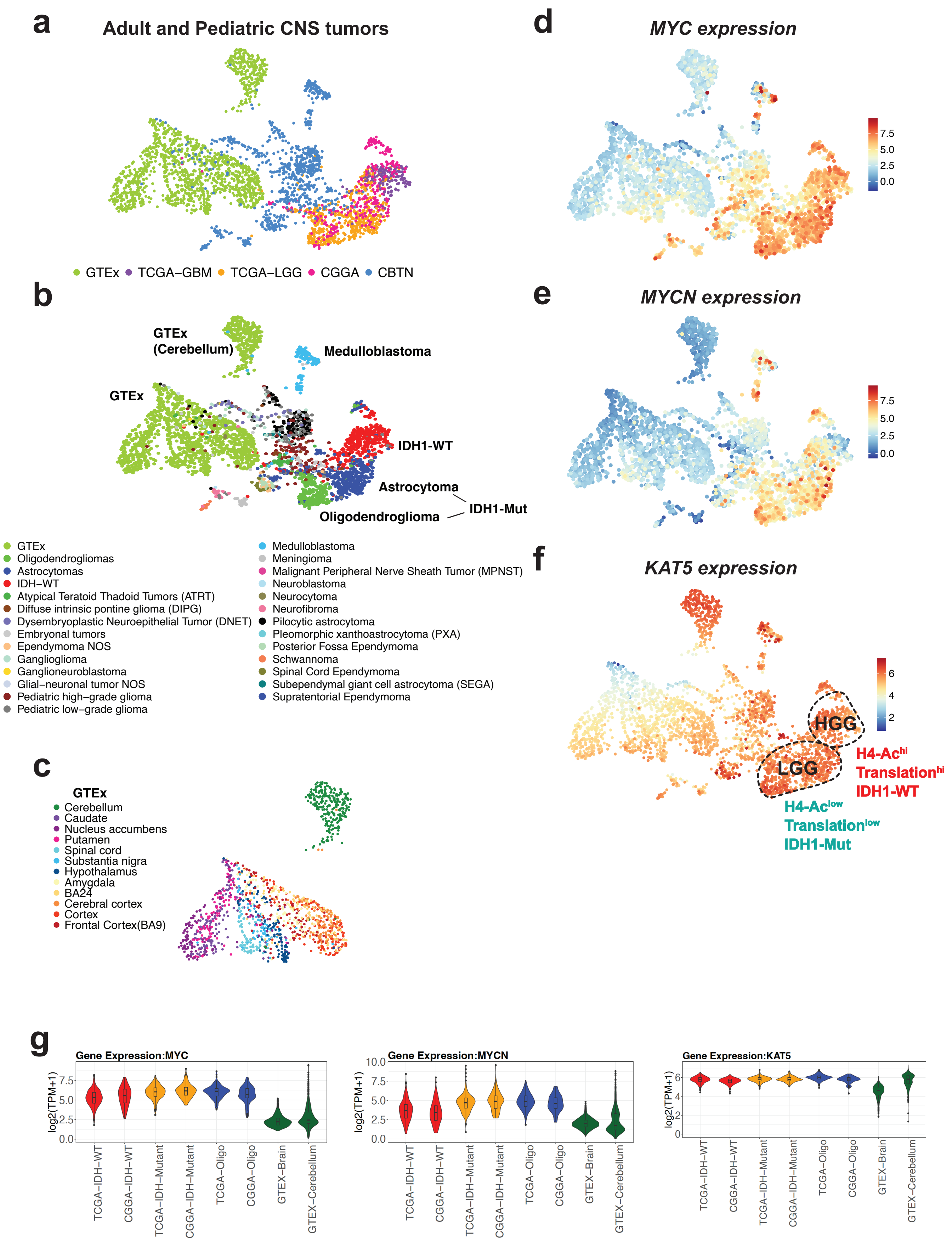

Supplementary Figure 16: Data in support of **Figure 8**

a, UMAP projections of “bulk” gene expression data from brain tissue and brain tumor samples reproduced from (PMID: 36918656) showing sample source. Tumors and tissue samples colored by study. CBTN= Children’s brain tumor network; CGGA= Chinese Glioma Genome Atlas (adult); GTEX= The Genotype-Tissue Expression Project; HGG=High grade glioma (adult); LGG= Low grade glioma (adult); TCGA = The Cancer Genome Atlas.

b, Color coded normal and tumor samples from **a** showing pediatric tumors and three subtypes for the adult gliomas.

c, UMAP showing unique clustering of GTEx-defined brain-regions.

d, *MYC* gene expression among UMAP samples from **a**.

e, *MYCN* gene expression among UMAP samples from **a**.

f, *KAT5* gene expression among UMAP samples from **a**.

g, Violin plots showing expression *MYC*, *MYCN*, and *KAT5* in HGG IDH-wt, astrocytoma IDH-mut, oligodendroglioma IDH-mut and GTEx control tissues.

Supplementary Figure S17

a

Intact cell / debris discrimination gate | Singlet / doublet discrimination gate | Secondary singlet / doublet discrimination gate | Live / dead discrimination gate

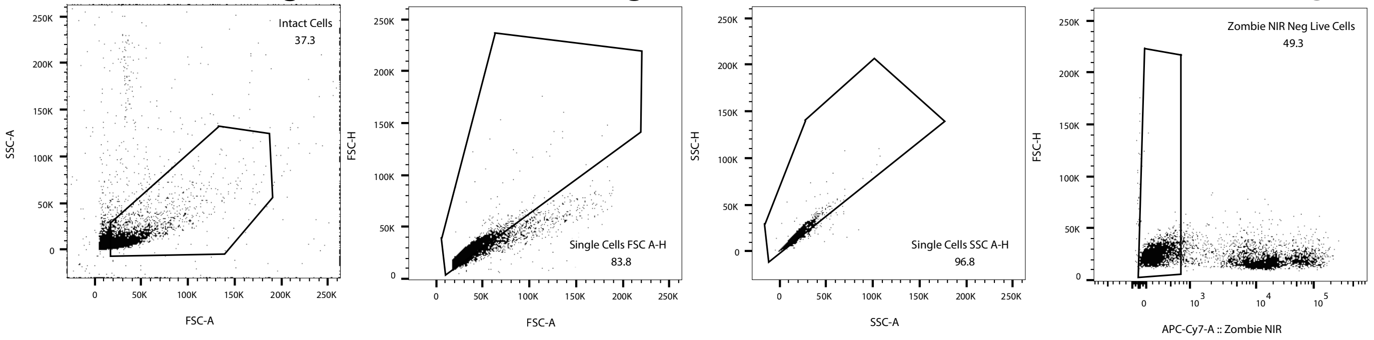

b

Immune cell discrimination gate | OPP vs H4Ac quadrant gate

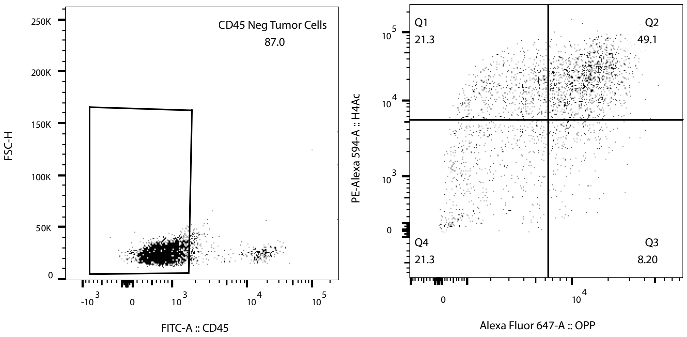

Supplementary Figure 17: General FACS gating strategies.
a, FACS gating strategies for analysis of live, intact, and single cells. For five/dead discrimination DAPI was used for live cell analyzed and fixable viability dye for samples that were fixed and further processed.
b, Additional gating strategies for FACS analysis of tumor cells in patient tumor samples used in **Fig. 7**. CD45 was used to eliminate immune cells.

Supplementary Figure S18

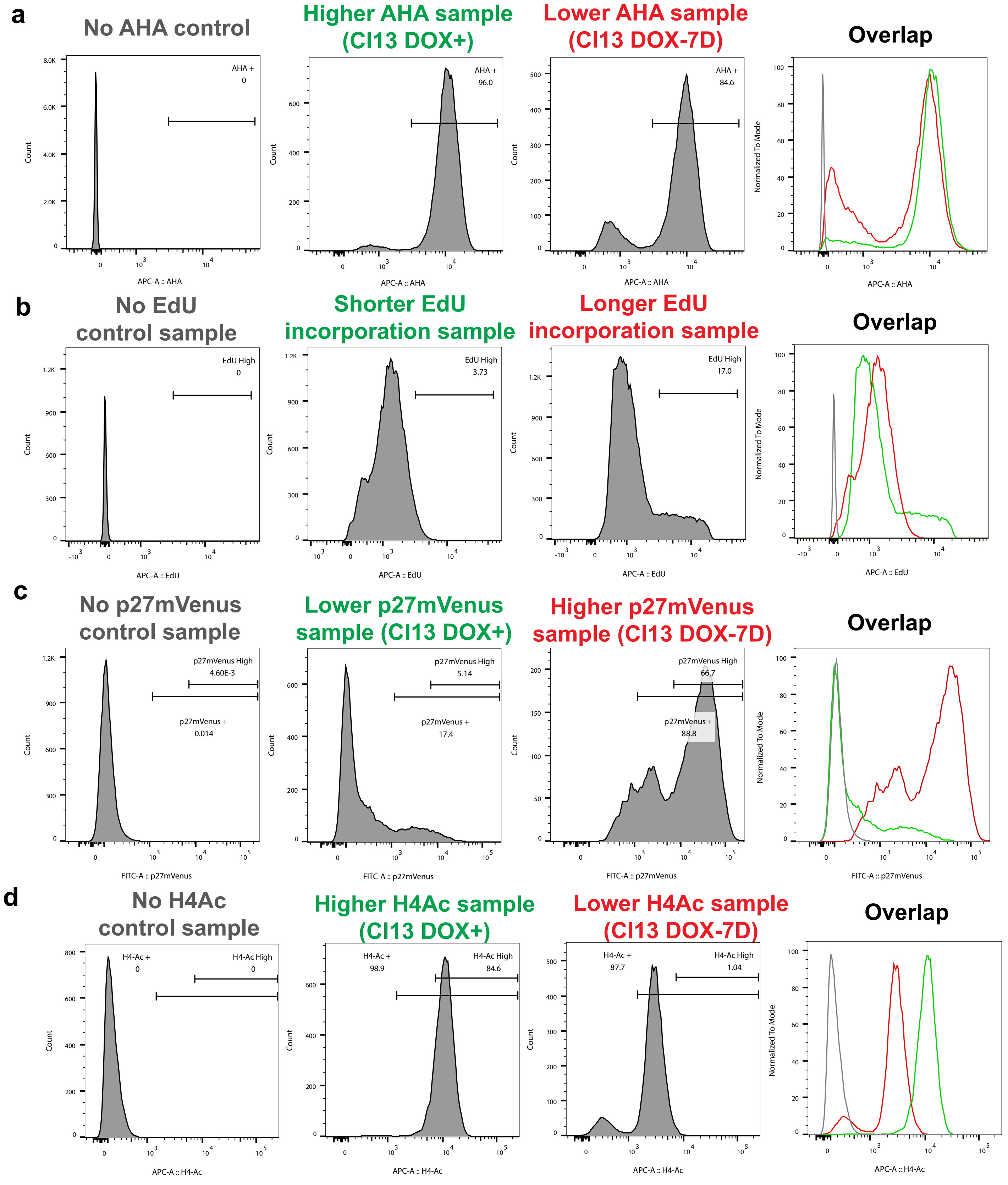

Supplementary Figure 18: FACS gating strategies for AHA, EdU, p27, and histone H4-Ac.
a, FACS gating strategies for AHA (and OPP) treated live cells.
b, FACS gating strategies for EdU treated live cells.
c, FACS gating strategies for p27-mVenus expressing live cells.
d, FACS gating strategies for histone H4-Ac staining of live cell.
