# Supplementary material for: KAT5 regulates neurodevelopmental states associated with G0-like populations in glioblastoma
Source: Nat Commun. 2025 May 9;16:4327. doi: 10.1038/s41467-025-59503-w (PMC12064679; doi:10.1038/s41467-025-59503-w)
Supplement: Supplementary file 2 — Description of Additional Supplementary Files [file 41467_2025_59503_MOESM2_ESM.pdf]

Description of Additional Supplementary Files

Supplementary Data 1: G0-trap screen results from GSC-0827 cells.

Supplementary Data 2: Gene expression analysis for *in vitro* KAT5 vs CD8 KO in GSC-0827.

Supplementary Data 3: Gene set enrichment analysis for top 200 expressed genes from each cluster for *in vitro* **Supplementary** KAT5 vs CD8 KO in GSC-0827, associated with **Figure 2**.

Supplementary Data 4: Gene set enrichment analysis for top 200 depleted genes from each cluster for *in vitro* KAT5 vs CD8 KO in GSC-0827, associated with **Figure 2**.

Supplementary Data 5: GBM gene expression modules used for scRNA-seq data analysis.

Supplementary Data 6: Analysis of KAT5 KO induced changes across 9 GSC isolates, associated with **Data 3** and **Supplementary Figure 6**.

Supplementary Data 7: Gene expression analysis for GSC-0827 tumor reference clusters, associated with **Figure 4** and **Supplementary Figure 8**.

Supplementary Data 8: Gene set enrichment analysis for top expressed genes for GSC-0827 tumor reference clusters, associated with **Figure 4** and **Supplementary Figure 8.**

Supplementary Data 9: Gene set enrichment analysis for top depleted genes for GSC-0827 tumor reference clusters **Figure 4** and **Supplementary Figure 8.**

Supplementary Data 10: Gene expression analysis for GSC-464T tumor reference clusters, associated with **Figure 4** and **Supplementary Figure 11**.

Supplementary Data 11: KAT5 binding sites in C13 tumors, associated with **Figure 5a**.

Supplementary Data 12: Full Venn diagram gene lists associated with **Figure 5c** and screen results for essential transcription factors in GSC-0827 cells.

Supplementary Data 13: ChromHMM analysis of H3K4me2, H3K27ac, and H3K27me3 marks in Dox+/KAT5+ GSC-0827 Dox-KAT5 tumors, in support of **Figure 5f**.

Supplementary Data 14: Differential Binding (DiffBind) analysis of H3K27ac marks in Dox+ versus Dox- GSC-0827 Dox-KAT5 tumors using DESeq2, in support of **Figure 5**.

Supplementary Data 15: Differential Binding (DiffBind) analysis of H3K27me3 marks in Dox+ versus Dox- GSC-0827 Dox-KAT5 tumors using DESeq2, in support of **Figure 5**.

Supplementary Data 16: Transcription factor binding sites predicted by Homer for genomic regions associated with significantly scoring changes in H3K27ac and H3K27me3 marks from **Supplementary Tables 14-15**, in support of **Supplementary Figure 12**.

Supplementary Data 17: Super enhancers predicted for $KAT5^{on}$ and $KAT5^{off}$ GSC-0827 tumors, in support of **Supplementary Figure 12**.

Supplementary Data 18: Description of glioma tumor samples used for **Fig. 7** and **Supplementary Figure 15**.

Supplementary Data 19: Key Resources
