# Supplementary material for: KAT5 regulates neurodevelopmental states associated with G0-like populations in glioblastoma
Source: Nat Commun. 2025 May 9;16:4327. doi: 10.1038/s41467-025-59503-w (PMC12064679; doi:10.1038/s41467-025-59503-w)
Supplement: Supplementary file 22 — Reporting Summary [file 41467_2025_59503_MOESM22_ESM.pdf]

nature portfolio

Corresponding author(s): Patrick J. Paddison, Anoop P. Patel

Last updated by author(s): Apr 15, 2025

Reporting Summary

Nature Portfolio wishes to improve the reproducibility of the work that we publish. This form provides structure for consistency and transparency in reporting. For further information on Nature Portfolio policies, see our Editorial Policies and the Editorial Policy Checklist.

Statistics

For all statistical analyses, confirm that the following items are present in the figure legend, table legend, main text, or Methods section.

n/a	Confirmed	
☐	☒	The exact sample size (*n*) for each experimental group/condition, given as a discrete number and unit of measurement
☐	☒	A statement on whether measurements were taken from distinct samples or whether the same sample was measured repeatedly
☐	☒	The statistical test(s) used AND whether they are one- or two-sided<br>*Only common tests should be described solely by name; describe more complex techniques in the Methods section.*
☒	☐	A description of all covariates tested
☒	☐	A description of any assumptions or corrections, such as tests of normality and adjustment for multiple comparisons
☐	☒	A full description of the statistical parameters including central tendency (e.g. means) or other basic estimates (e.g. regression coefficient) AND variation (e.g. standard deviation) or associated estimates of uncertainty (e.g. confidence intervals)
☐	☒	For null hypothesis testing, the test statistic (e.g. *F*, *t*, *r*) with confidence intervals, effect sizes, degrees of freedom and *P* value noted<br>*Give P values as exact values whenever suitable.*
☒	☐	For Bayesian analysis, information on the choice of priors and Markov chain Monte Carlo settings
☒	☐	For hierarchical and complex designs, identification of the appropriate level for tests and full reporting of outcomes
☒	☐	Estimates of effect sizes (e.g. Cohen's *d*, Pearson's *r*), indicating how they were calculated

Our web collection on statistics for biologists contains articles on many of the points above.

Software and code

Policy information about availability of computer code

Data collection	SG Cell Line 96-well Nucleofector Kit Lonza Cat# V4SC-3096<br>Click-iT Plus kit Thermo Fisher Cat# C10643<br>Brain tumor dissociation kit Miltenyi Cat# 130-095-942<br>CUTANA CUT&Tag kit EpiCypher Cat# 14-1101<br>Chromium Next GEM Single Cell 3’ Kit v3.1 10X Genomics Cat# PN-1000269<br>BD FACSDiva v9.0 BD Biosciences https://www.bdbiosciences.com/en-us/products/software/instrument-software/bd-facsdiva-software<br>Image Studio Li-Cor https://www.licor.com/bio/image-studio/<br>CUT&Tag and scRNA-seq data was generated and sequenced at Fred Hutch Cancer Center
Data analysis	The single-cell RNA sequencing data files are available on the GEO database at GSE198524. [Review token access : wtehwmkonvypbyv].<br>The code used to process and analyze the data is available at https://github.com/sonali bioc/GSC_scRNASeq_KAT5paper.<br>CellRanger (v5.0 from 10× Genomics) was used to align, quantify, and provide basic quality control metrics for the scRNA-seq data. Souporcell was used to demultiplex scRNA-seq data for each GSC cell line. Using Seurat (version 4), the scRNA-seq data was normalized using the SCTransform pipeline and were merged the GSC 827 tumor replicates to build an integrated reference. FindTransferAnchors and MapQuery from Seurat, was used to map the query tumors to the integrated reference. FindAllMarkers was used to find differentially expressed genes for each cluster of each tumor. AddModuleScore from Seurat was used to calculate the average expression levels of different gene lists of interest for each tumor type. ggplot2 was used to make bar plots to visualize the number of genes and cells in each cluster. ccSeurat and ccAF were used to score cell cycle states for each cell. scVelo was used to perform velocity analysis. The ToppCell Atlas was used to perform gene set enrichment analysis on each of the differentially expressed gene list from each cluster. CUT&Tag data analysis: genomes were aligned using bowtie2 with the following parameters: --end-to-end --very-sensitive --no-mixed --no-discordant --phred33 -I 10 -X 700.

Reads were sorted and indexed using Samtools. Coverage files, matrices, and heatmaps were generated using DeepTools. Chromatin states were defined by ChromHMM analysis of the .bam alignment files.
GraphPad Prism version 9.0 GraphPad Software https://www.graphpad.com/scientific-software/prism/
FlowJo version 10.7.2 FlowJo Software https://www.flowjo.com/
Horos Project Horors Dicom Viewer Software horosproject.org
Microsoft Excel Excel Software https://www.microsoft.com/en-us/microsoft-365/excel
Cell Ranger v5.0 10x Genomics https://support.10xgenomics.com/single-cell-gene-expression/software/downloads/latest
ChromHMM_CC Ernst and Kellis, 2012 http://compbio.mit.edu/ChromHMM
HOMER_CC Heinz et al, 2010 http://homer.ucsd.edu/homer/
Diffbind_CC Ross-Innes et al, 2011 http://bioconductor.org/packages/release/bioc/html/DiffBind.html
FIJI NIH https://imagej.net/software/fiji/
Image Studio Li-Cor https://www.licor.com/bio/image-studio/
R (v.3.3.1), RStudio (v.1.0.136) Posit, PBC https://www.r-project.org/
Bowtie2: https://bowtie-bio.sourceforge.net/bowtie2/index.shtml
Samtools: http://www.htslib.org/
https://deeptools.readthedocs.io/en/latest/

For manuscripts utilizing custom algorithms or software that are central to the research but not yet described in published literature, software must be made available to editors and reviewers. We strongly encourage code deposition in a community repository (e.g. GitHub). See the Nature Portfolio guidelines for submitting code & software for further information.

Data

Policy information about availability of data

All manuscripts must include a data availability statement. This statement should provide the following information, where applicable:
- Accession codes, unique identifiers, or web links for publicly available datasets
- A description of any restrictions on data availability
- For clinical datasets or third party data, please ensure that the statement adheres to our policy

The single-cell RNA sequencing data files are available on the GEO database at GSE198524. [Review token access : wtehwmkonvypbyv].The code used to process and analyze the data is available at https://github.com/sonalibioc/GSC_scRNASeq_KAT5paper; https://github.com/plaisier-lab/KAT5paper

Research involving human participants, their data, or biological material

Policy information about studies with human participants or human data. See also policy information about sex, gender (identity/presentation), and sexual orientation and race, ethnicity and racism.

Reporting on sex and gender	All relevant info is described in Supplementary Data 18.
Reporting on race, ethnicity, or other socially relevant groupings	All relevant info is described in Supplementary Data 18.
Population characteristics	All relevant info is described in Supplementary Data 18.
Recruitment	Over the course of 2 years, 10 patient glioma tumors were collected and assayed for H4 acetylation levels and protein synthesis rates: 3 LGGs (UW33, UW36, UW44), 5 HGGs (UW27, UW31, UW34, UW38, UW40), and 2 HGGs IDH1/2 mutant (UW26, UW45), which represent recurrent LGGs.
Ethics oversight	Patient glioma samples were obtained under Institutional Review Board (IRB)-approved protocols from University of Washington Medical Center in accordance with national guidelines. All patients signed informed consent during clinical visits before surgery and sample collection.

Note that full information on the approval of the study protocol must also be provided in the manuscript.

Field-specific reporting

Please select the one below that is the best fit for your research. If you are not sure, read the appropriate sections before making your selection.

☒ Life sciences ☐ Behavioural & social sciences ☐ Ecological, evolutionary & environmental sciences

For a reference copy of the document with all sections, see nature.com/documents/nr-reporting-summary-flat.pdf

Life sciences study design

All studies must disclose on these points even when the disclosure is negative.

Sample size	Sample size was determined based on statistical significance. Some sample sizes were limited on sample availability (glioma patient samples), and rare specimen and expensive reagents (some in vivo data, sc-RNA-seq).
Data exclusions	We excluded a replicate of H3K4me2 CUT&Tag due to failure.

	The tumor volume data from Figure 6g was restricted to 6 weeks post SOC treatments since at later time points some mice died in the absence of post-treatment tumor recurrence.
Replication	The majority of experiments were performed at least in triplicates and measurements were reproducible. Replicates of patient glioma samples depended on sample availability. CUT&Tag data was duplicated for each of the 3 marks. The sc-RNA seq and tumor volumetric experiments for KAT5 titration experiments were not replicated. For survival and tumor volumetrics in KAT-KO and/or Standard of Care at least 5 mice were enrolled in each experimental cohort.
Randomization	No randomization was done.
Blinding	Experimenters were not blinded to experimental conditions.

Reporting for specific materials, systems and methods

We require information from authors about some types of materials, experimental systems and methods used in many studies. Here, indicate whether each material, system or method listed is relevant to your study. If you are not sure if a list item applies to your research, read the appropriate section before selecting a response.

Materials & experimental systems

n/a	Involved in the study	
☐	☒	Antibodies
☐	☒	Eukaryotic cell lines
☒	☐	Palaeontology and archaeology
☐	☒	Animals and other organisms
☒	☐	Clinical data
☒	☐	Dual use research of concern
☒	☐	Plants

Methods

n/a	Involved in the study	
☒	☐	ChIP-seq
☐	☒	Flow cytometry
☐	☒	MRI-based neuroimaging

Antibodies

Antibodies used

Name Source Product number
IRDye 680RD Goat anti-Rabbit IgG (2° WB, 1:10000) Licor Cat# 925-68071
IRDye 800CW Donkey anti-Goat IgG (2° WB, 1:10000) Licor Cat# 926-32214
IRDye 800CW Goat anti-Mouse IgG (2° WB, 1:10000) Licor Cat# 926-32210
anti-Rabbit AF647 (2° FC, 1:200) Thermo Fisher Scientific Cat# A-21245, RRID:AB_2535813
anti-Mouse AF488 (2° FC, 1:200) Thermo Fisher Scientific Cat# A-11001, RRID:AB_2534069
Anti-Mouse (2° CUT&Tag 1:100) EpiCypher Cat# 13-0048
Anti-Rabbit (2° CUT&Tag 1:100) Antibodies Online Cat# ABIN101961
anti-Rabbit AF568 (2° FC, 1:200) Thermo Fisher Scientific Cat# A-11011, RRID:AB_143157
anti-Beta-Actin (WB,1:1000) Cell Signaling Technology Cat# 3700, RRID:AB_2242334
anti-Actin (WB, 1:150) DSHB Cat# JLA20
anti-Alpha Tubulin (WB, 1:1000) Sigma Cat# T9026
anti-Histone H3 (mAbcam 24834) (WB 1:2000) Abcam Cat# 24834
Histone H4K12ac Antibody (WB, 1:1000) EpiCypher Cat# 13-0037
Histone H4K20ac Antibody (WB, 1:1000) EpiCypher Cat# 13-0039
Histone H4K8ac Antibody (WB, 1:1000) EpiCypher Cat# 13-0036
Histone H4ac (pan-acetyl) (WB, 1:1000; FC, 1:100) ActiveMotif Cat# 39026
Histone H3K27me3 Antibody (WB, 1:1000, CUT&Tag, 1:100) Cell Signaling Technologies Cat# 9733
Histone H3K27ac Antibody (WB, 1:1000, CUT&Tag, 1:100) Millipore Cat# MABE647
Histone H3K4Me2 (CUT&Tag 1:100) Millipore Cat# 07-030
Histone H4K16ac Antibody (WB, 1:1000) Serotec Cat# AHP417
Histone H4K20me3 (WB, 1:1000) EpiGentek Cat# A-4048-025
anti-PTN (WB, 1:250) SCBT Cat# sc-74443
anti-SPARCL1 (WB, 1:200) Fisher Cat# AF2728SP
anti-RPTPbeta antibody (PTPRZ1) (WB, 1:500) BD Biosciences Cat# BDB610179
anti-S100B (E7C3A) (WB, 1:400) Cell Signaling Technology Cat# 90393
anti-SPARC (WB, 1:200) DSHB Cat# SPARC MAB 303
anti-MYC (WB, 1:2000) Cell Signaling Technology Cat# 13987
anti-AURKA (WB, 1:1000) Cell Signaling Technology Cat# 4718
anti-KAT5 (WB, 1:1000) Cell Signaling Technology Cat# 12058
anti-p27 Kip1 (D69C12) (WB, 1:1000) Cell Signaling Technology Cat# 3686
anti-CD45 (HI30) (FC, 1:20) Fisher Scientific Cat# 509591
anti-CD142 (F3) (FC, 1:11) Miltenyi Biotec Cat#130098745
anti-V5 (CUT&Tag 1:25) Invitrogen Cat# R960-25

Validation	Antibodies used in this study were picked according to the manufacturer's validations for the species and the application used here. The H4-pan Ac antibody used for Flow Cytometry was validated by using different titers and negative controls and by comparisons to WB results using the same samples and antibody.

Eukaryotic cell lines

Policy information about cell lines and Sex and Gender in Research

Cell line source(s)	GSC-0131 Human GBM isolate Son et al., 2009; PMID: 19427293<br>GSC-0827 Human GBM isolate Son et al., 2009; PMID: 19427293<br>GSC-0308 Human GBM isolate Son et al., 2009; PMID: 19427293<br>GSC-448T Human GBM isolate Joo et al., 2013; PMID: 23333277<br>GSC-464T Human GBM isolate Joo et al., 2013; PMID: 23333277<br>GSC-578T Human GBM isolate Joo et al., 2013; PMID: 23333277<br>GSC-025T Human GBM isolate Joo et al., 2013; PMID: 23333277<br>GSC-1406 Human GBM isolate PMID: 31693904<br>GSC-G7 Human GBM isolate Al-Mayhani et al., 2009; PMID: 19215724<br>293T Human embryonic kidney cell line ATCC Cat#: CRL-3216; RRID:CVCL_0063
Authentication	GBM isolates were authenticated by exome sequencing.
Mycoplasma contamination	GSC-0827, GSC-0308, GSC-446T, GSC-448T, GSC-025T, GSC-1406, GSC-G7 and GSC-0131 tested negative for Mycoplasma contamination. The other cell lines used here were not tested for mycoplasma.
Commonly misidentified lines<br>(See ICLAC register)	None

Animals and other research organisms

Policy information about studies involving animals; ARRIVE guidelines recommended for reporting animal research, and Sex and Gender in Research

Laboratory animals	NSG mice (Jackson Labs #005557) were used starting from 6 weeks of age.
Wild animals	None
Reporting on sex	Mice of both sexes were used in this study.
Field-collected samples	None
Ethics oversight	All animal experimental procedures were performed with approval of the FHCC Institutional Animal Care and Use Committee. All procedures followed guidelines outlined in the National Research Council Guide for the Care and Use of Laboratory Animals.

Note that full information on the approval of the study protocol must also be provided in the manuscript.

Flow Cytometry

Plots

Confirm that:

- [ ] The axis labels state the marker and fluorochrome used (e.g. CD4-FITC).
- [x] The axis scales are clearly visible. Include numbers along axes only for bottom left plot of group (a 'group' is an analysis of identical markers).
- [ ] All plots are contour plots with outliers or pseudocolor plots.
- [ ] A numerical value for number of cells or percentage (with statistics) is provided.

Methodology

Sample preparation	Glioma samples from patients and GSC-derived mouse brain xenografts were dissociated using Miltenyi (130-095-942) brain tumor dissociation kit. GSC adherent cultures cells were dissociated by Accutase. Dissociated cells were then incubated with the experimental reagents/stains/antibodies and cells were resuspended in FACS buffer for analysis/sorting. Glioma patient derived cells were counted, and while alive, they were incubated with OPP (2 µM final, 1 million cells in 1 ml NSC media, in a low binding tube at 37oC for 30 min). Then cells were washed in warm NSC, and slowly frozen (1oC cooling/1 min.) in NSC media supplemented with 10% DMSO. Cohorts of HGG WT and LGG and/or HGG MUT were processed together in order to use the HGG WT as normalization among cohorts that were collected over 2 years. Frozen cells were thawed using a method that recovers a large percentage of viable cells after freeze/thaw (frozen cells were thawed at 37oC for 2 min., then warm NSC media was added dropwise to cells by doubling the volume every minute to a total volume of 32 ml, starting from one 1ml frozen cell vial). While cells were alive, a CD45 antibody was used to surface stain immune tumor populations, a

	viability fixable Zombie dye (BioLegend) was used to determine live cells, then fixed in 4% PFA, permeabilized, and processed for Click-iT chemistry to detect OPP, and intracellularly stained for H4Ac. Processed cells were flow cytometry analyzed immediately.
Instrument	FACS cell analyzers used in this study were: BD FACSymphony or BD LSRFortessa X-50<br>FACS cell analyzers used in this study were: BD FACSAria II, BD FACSymphony S6 or Sony MA900.
Software	For acquisition we used BD or Sony FACS softwares from the instruments used for each experiment, and FlowJo software was used for analysis.
Cell population abundance	For patient-derived glioma samples the dissociated cells had viability between 49 and 96% according to exclusion of the fixable Zombie viability dye (Biolegend), and glioma purity in the live cells was between 46 and 98% according to exclusion of the immune populations that were stained using CD45 antibody
Gating strategy	All samples were gated based on FSC/SSC to exclude cellular debris, aggregates and dead cells. For primary glioma samples a gate on Zombie dye negative cells was used to isolate live cells, followed by a CD45 negative gate to isolate the non-immune tumor cell populations that were entered into the downstream analysis to evaluate protein synthesis rates (OPP+), and KAT5 activity (H4Ac+).

[x] Tick this box to confirm that a figure exemplifying the gating strategy is provided in the Supplementary Information.

Magnetic resonance imaging

Experimental design

Design type	GSC-derived brain mouse xenografts were blocked imaged by MRI to determine tumor volume dependence to KAT5 protein titration and standard of care treatments.
Design specifications	After a tumor size of about 2 mm^3 was verified by MRI, the mice were given different Doxycycline concentrations in their drinking water in order to manipulate the KAT5 protein titers and/or were treated with standard of care, and MRI was repeated with a frequency of once/twice per week.
Behavioral performance measures	N/A

Acquisition

Imaging type(s)	Structural
Field strength	1T, 7T
Sequence & imaging parameters	T1- weighted, T2-weighted
Area of acquisition	Frontal brain

Diffusion MRI [ ] Used [x] Not used

Preprocessing

Preprocessing software	N/A
Normalization	N/A
Normalization template	N/A
Noise and artifact removal	N/A
Volume censoring	N/A

Statistical modeling & inference

Model type and settings	N/A
Effect(s) tested	N/A

Specify type of analysis: [x] Whole brain [ ] ROI-based [ ] Both

Statistic type for inference<br>(See Eklund et al. 2016)	N/A
Correction	N/A

Models & analysis

n/a	Involved in the study
☒	☐ Functional and/or effective connectivity
☒	☐ Graph analysis
☒	☐ Multivariate modeling or predictive analysis
